# Comparison of 4-Factor Prothrombin Complex Concentrate With Frozen Plasma for Management of Hemorrhage During and After Cardiac Surgery: A Randomized Pilot Trial

**DOI:** 10.1001/jamanetworkopen.2021.3936

**Published:** 2021-04-01

**Authors:** Keyvan Karkouti, Justyna Bartoszko, Deep Grewal, Cielo Bingley, Chantal Armali, Jo Carroll, Hans-Peter Hucke, Amie Kron, Stuart A. McCluskey, Vivek Rao, Jeannie Callum

**Affiliations:** 1 Department of Anesthesia and Pain Management; University Health Network, Sinai Health System, Women’s College Hospital, University of Toronto, Toronto, Ontario, Canada; 2 Peter Munk Cardiac Centre and Toronto General Hospital Research Institute, University Health Network, Toronto, Ontario, Canada; 3 Department of Laboratory Medicine and Molecular Diagnostics, Sunnybrook Health Sciences Centre, Toronto, Ontario, Canada; 4 Department of Biostatistics, Ergomed CDS GmbH, Cologne, Germany; 5 Division of Cardiovascular Surgery, Toronto General Hospital, University Health Network, University of Toronto, Toronto, Ontario, Canada; 6 Department of Laboratory Medicine and Pathobiology, University of Toronto, Toronto, Ontario, Canada; 7 Department of Pathology and Molecular Medicine, Kingston Health Sciences Centre and Queen’s University, Kingston, Ontario, Canada

## Abstract

**Importance:**

Approximately 15% of patients undergoing cardiac surgery receive frozen plasma (FP) for bleeding. Four-factor prothrombin complex concentrates (PCCs) have logistical and safety advantages over FP and may be a suitable alternative.

**Objectives:**

To determine the proportion of patients who received PCC and then required FP, explore hemostatic effects and safety, and assess the feasibility of study procedures.

**Design, Setting, and Participants:**

Parallel-group randomized pilot study conducted at 2 Canadian hospitals. Adult patients requiring coagulation factor replacement for bleeding during cardiac surgery (from September 23, 2019, to June 19, 2020; final 28-day follow-up visit, July 17, 2020). Data analysis was initiated on September 15, 2020.

**Interventions:**

Prothrombin complex concentrate (1500 IU for patients weighing ≤60 kg and 2000 IU for patients weighing >60 kg) or FP (3 U for patients weighing ≤60 kg and 4 U for patients weighing >60 kg), repeated once as needed within 24 hours (FP used for any subsequent doses in both groups). Patients and outcome assessors were blinded to treatment allocation.

**Main Outcomes and Measures:**

Hemostatic effectiveness (whether patients received any hemostatic therapies from 60 minutes to 4 and 24 hours after initiation of the intervention, amount of allogeneic blood components administered within 24 hours after start of surgery, and avoidance of red cell transfusions within 24 hours after start of surgery), protocol adherence, and adverse events. The analysis set comprised all randomized patients who had undergone cardiac surgery, received at least 1 dose of either treatment, and provided informed consent after surgery.

**Results:**

Of 169 screened patients, 131 were randomized, and 101 were treated (54 with PCC and 47 with FP), provided consent, and were included in the analysis (median age, 64 years; interquartile range [IQR], 54-73 years; 28 [28%] were female; 82 [81%] underwent complex operations). The PCC group received a median 24.9 IU/kg (IQR, 21.8-27.0 IU/kg) of PCC (2 patients [3.7%; 95% CI, 0.4%-12.7%] required FP). The FP group received a median 12.5 mL/kg (IQR, 10.0-15.0 mL/kg) of FP (4 patients [8.5%; 95% CI, 2.4%-20.4%] required >2 doses of FP). Hemostatic therapy was not required at the 4-hour time point for 43 patients (80%) in the PCC group and for 32 patients (68%) in the FP group (*P* = .25) nor at the 24-hour time point for 41 patients (76%) in the PCC group and for 31 patients (66%) patients in the FP group (*P* = .28). The median numbers of units for 24-hour cumulative allogeneic transfusions (red blood cells, platelets, and FP) were 6.0 U (IQR, 4.0-11.0 U) in the PCC group and 14.0 U (IQR, 8.0-20.0 U) in the FP group (ratio, 0.58; 95% CI, 0.45-0.77; *P* < .001). After exclusion of FP administered as part of the investigational medicinal product, the median numbers of units were 6.0 U (IQR, 4.0-11.0 U) in the PCC group and 10.0 U (IQR, 6.0-16.0 U) in the FP group (ratio, 0.80; 95% CI, 0.59-1.08; *P* = .15). For red blood cells alone, the median numbers were 1.5 U (IQR, 0.0-4.0 U) in the PCC group and 3.0 U (IQR, 1.0-5.0 U) in the FP group (ratio, 0.69; 95% CI, 0.47-0.99; *P* = .05). During the first 24 hours after start of surgery, 15 patients in the PCC group (28%) and 8 patients in the FP group (17%) received no red blood cells (*P* = .24). Adverse event profiles were similar.

**Conclusions and Relevance:**

This randomized clinical trial found that the study protocols were feasible. Adequately powered randomized clinical trials are warranted to determine whether PCC is a suitable substitute for FP for mitigation of bleeding in cardiac surgery.

**Trial Registration:**

ClinicalTrials.gov Identifier: NCT04114643

## Key Points

**Question:**

Can 4-factor prothrombin complex concentrate (PCC) be used as a substitute for frozen plasma (FP) for treatment of bleeding related to coagulation factor deficiency in cardiac surgery?

**Findings:**

In this pilot trial, 101 adult patients were successfully randomized to receive PCC or FP for bleeding during cardiac surgery. In exploratory analyses, the PCC group had significantly lower chest tube blood loss and received fewer allogeneic blood transfusions, whereas the number of thromboembolic events was similar.

**Meaning:**

Four-factor PCC may be a suitable alternative to FP for management of cardiac surgery–associated coagulopathy, pending assessment by adequately powered multicenter trials.

## Teaser

This parallel-group randomized pilot study assesses whether 4-factor prothrombin complex concentrate can substitute for frozen plasma in the treatment of bleeding related to coagulation factor deficiency in adult patients during and after cardiac surgery.

## Introduction

Cardiac surgery imposes multiple stressors on the coagulation system, resulting in systemic derangements that include the depletion of enzymatic coagulation factors to a degree that impairs thrombin generation and may lead to excessive bleeding and transfusion.^1^ Sufficient amounts of activated thrombin are needed to convert soluble fibrinogen into insoluble strands of fibrin (thereby forming a fibrin clot) and to catalyze multiple other coagulation-related reactions.^2,3^ Impaired thrombin generation is an important cause of coagulopathy and excessive bleeding in cardiac surgery.^4^ Consequently, replenishment of coagulation factors is an important aspect of a multimodal approach to perioperative coagulopathy.^5,6^

To replenish depleted coagulation factors and thereby improve thrombin generation in bleeding patients, 2 therapeutics, frozen plasma (FP) and prothrombin complex concentrate (PCC), are available. Frozen plasma, which contains the full complement of procoagulant and anticoagulant factors that are present in blood, has long been the mainstay of therapy for this purpose. In the United States, FP is administered to approximately 15% of all cardiac surgery patients and to one-third of all bleeding patients.^7,8^ This widespread use exists despite the lack of data supporting the effectiveness of FP^9,10^ and its potential for causing major complications, such as transfusion-related acute lung injury and transfusion-associated circulatory overload.^11^

Purified from pooled plasma and widely available, PCCs offer a potential alternative to FP for management of bleeding. Although specific compositions vary depending on the manufacturer, 4-factor PCCs contain vitamin K–dependent coagulation factors (factors II, VII, IX, and X), the anticoagulant proteins C and S (some also contain antithrombin), and small amounts of heparin.^12^ The advantages of PCCs relative to FP are that they are pathogen reduced, do not require ABO blood group matching or thawing (allowing for near-patient storage and timely administration), are manufactured from pooled plasma (decreasing the risk of transfusion-related acute lung injury), require substantially lower volumes to achieve dose equivalence with FP (decreasing the risk of transfusion-associated circulatory overload), and may lead to greater thrombin generation (improving hemostatic effectiveness).^12,13,14,15^ On the other hand, because PCCs do not contain the full, balanced complement of procoagulants and anticoagulants that are present in FP, they may be less effective in restoring hemostasis, may carry a higher thrombosis risk (possibly owing to an imbalance in factor II to antithrombin ratio), or both.^12,16^

Several high-quality randomized clinical trials have shown that PCCs are superior to FP for reversal of vitamin K antagonists, and they are now the agent of choice for that indication.^17^ However, to our knowledge, large-scale comparative randomized clinical trials assessing the broader use of PCCs for treatment of coagulopathy due to coagulation factor deficiency in cardiac surgery have not been conducted. In preparation for such a study, we conducted this randomized pilot trial comparing PCC with FP in patients undergoing cardiac surgery who were bleeding and required coagulation factor replenishment to determine the proportion of patients who first received PCC but also required FP, to compare the hemostatic effects and safety of the 2 therapeutics, and to assess the feasibility of study procedures.

## Methods

### Trial Oversight

This was an investigator-initiated, randomized clinical trial conducted at 2 centers in Canada (Sunnybrook Health Sciences Centre and Toronto General Hospital). The trial is registered at ClinicalTrials.gov^18^ and was conducted by the Anesthesia Clinical Trials Unit at the University Health Network (Toronto, Ontario, Canada). The trial was overseen by an independent data and safety monitoring committee, and study monitors independently reviewed all primary outcomes and adverse events. The trial was performed in accordance with the principles of the Declaration of Helsinki^19^ and applicable regulatory requirements. The trial protocol and statistical analysis plan are provided in Supplement 1. This study followed the Consolidated Standards of Reporting Trials (CONSORT) reporting guideline. Research ethics board approval was obtained at each site before trial initiation, with permission to obtain written informed consent from patients or surrogates as soon as possible after surgery. All participants or their surrogates provided written informed consent unless otherwise indicated, and no one received compensation or was offered any incentive for participating in this study.

### Patients

Adult patients undergoing cardiac surgery for whom coagulation factor replacement with FP or PCC was ordered during surgery for management of bleeding were eligible for inclusion. Blood bank technologists screened (by telephone, in collaboration with the operating team) and randomized eligible patients if they had none of the following exclusion criteria: receipt of FP or PCC within 48 hours before surgery; history of severe allergic reaction to FP or PCC; refusal of blood components; known pregnancy; anticipated high risk of death within 24 hours of surgery; undergoing heart transplantation, ventricular assist device implant or removal, or thoracoabdominal aneurysm repair; history of heparin-induced thrombocytopenia; receipt of warfarin with an international normalized ratio higher than 1.5 at the time of surgery; or receipt of direct oral anticoagulants within 48 hours of surgery.

### Trial Procedures

Participants were randomly assigned (1:1 ratio) to study groups using a pseudorandom number generator (PROC PLAN procedure in SAS) in randomly permuted blocks of 4, stratified by center. Allocation was blinded; the randomization schedule was kept at the blood banks in sequentially numbered opaque sealed envelopes (prepared by Ergomed GmbH), which were opened when the order for PCC or FP was received. For the first and second orders up to 24 hours after randomization, patients assigned to the PCC group received Octaplex (Octapharma AG), 1500 IU if the patient weighed 60 kg or less or 2000 IU if the patient weighed more than 60 kg, and those assigned to the FP group received FP (Canadian Blood Services), 3 U if the patient weighed 60 kg or less or 4 U if the patient weighed more than 60 kg (each unit approximately 250 mL) for each order. For any additional orders, FP was administered to both groups.

Given that the products have different physical properties, it was not possible to blind treating clinicians to group assignment. To minimize bias, the first set of products was released in weight-matched, tamper-sealed containers that were opened immediately before initiating treatment, thereby ensuring that clinicians remained blinded to group allocation until after the decision was made to administer the investigational product. Clinicians not involved in product administration, patients, family members, and all study personnel remained blinded to group assignment. Medical record labels for both products stated “FARES Study Product 1 U.”

There were no other alterations to patient care. Both hospitals used a transfusion algorithm that used point-of-care and standard coagulation assays and recommended a targeted, stepwise approach for management of bleeding (recommended order: platelets, fibrinogen concentrate, and FP or PCC).^20^ Administration of hemostatic adjuncts and cell salvage was conducted according to hospital practice. Tranexamic acid was administered prophylactically to all patients. Protocol deviations were classified as major if inclusion or exclusion criteria were violated, an unassigned investigational product was administered, or less than 80% of the assigned first dose was administered.

### Outcome Measures

The primary measures of hemostatic effects were (1) treatment response, based on receipt of any hemostatic therapies from 60 minutes to 4 and 24 hours after initiation of the intervention; (2) cumulative and individual allogeneic blood component units (red blood cells, platelets, and FP) administered within 24 hours after start of surgery (for platelets, both standard buffy coat pools from 4 allogeneic donors and 1 apheresis unit from a single allogeneic donor were counted as a 4-U transfusion); and (3) avoidance of red cell transfusion within 24 hours after start of surgery. Other measures of hemostatic effects included cumulative and individual allogeneic blood component units administered within 24 hours and 7 days after cardiopulmonary bypass and within 24 hours after start of intervention; blood loss, as measured by chest tube drainage at 12 and 24 hours after surgery; number of patients receiving hemostatic factor concentrates; and bleeding severity, as measured by the universal definition of perioperative bleeding score.^21^ The measures for assessing feasibility of study procedures were successful randomization, treatment according to group allocation, and attainment of informed consent after surgery. To assess the suitability of PCC as a substitute for FP, the number of patients in the PCC group who ultimately required FP was recorded.

### Sample Size Determination

No formal sample size calculation was carried out. It was deemed that a sample of 100 treated patients would be sufficient to assess the feasibility of study procedures, to determine the proportion of patients who received PCC and would require FP, and to determine event rates for other measured outcomes. Based on previous experience,^22^ it was anticipated that approximately 120 randomized patients would yield 100 treated patients.

### Statistical Analysis

Data analysis was initiated on September 15, 2020. All analyses followed the a priori defined statistical analysis plan, unless otherwise specified. The analysis set comprised all randomized patients who had undergone cardiac surgery, received at least 1 (partial or complete) dose of either treatment, and provided informed consent after surgery (either directly or via surrogate). Outcomes were examined using descriptive statistics, point estimates with 2-sided 95% CIs, the Fisher exact test, the *t* test, the Wilcoxon signed rank test, and negative binomial regression analysis as appropriate. Allogeneic blood component comparisons were based on the PCC to FP treatment group ratio of the least-squares mean number of units transfused. Comparisons were prespecified as superiority analyses with α = .05. Variables with missing values are noted when applicable, and patients with missing values were excluded from the relevant analyses. Analyses were performed using SAS, version 9.4 (SAS Institute Inc).

## Results

Patient recruitment continued from September 23, 2019, to June 19, 2020, with the final follow-up visit on July 17, 2020. The study was terminated when the prespecified number of evaluable patients was recruited, and no interim analyses were conducted. Of the 1360 patients who underwent cardiac surgery during the study period, coagulation factor replacement was requested for 169 patients (12.4%), and 131 patients (77.5%) were randomized (Figure). Of the randomized patients, 27 were not treated owing to cessation of bleeding at the time of product availability, 1 patient refused consent (and therefore was fully excluded from the analyses), and consent could not be obtained from 2 patients or their surrogate decision maker, leaving 101 patients in the final analysis set (54 in the PCC group and 47 in the FP group). Data on mortality and serious adverse events were collected from the 2 patients for whom consent could not be obtained (as per research ethics board approval). No patients in the analysis set had missing data on transfusions or adverse events; thus, they were all included in the relevant analyses. Major protocol deviations occurred for 15 patients (14.9%) (4 patients in the PCC group and 11 patients in the FP group). Except for 1 patient in the PCC group who received FP after only 1 dose of PCC was administered, all major deviations were for receiving less than 80% of the assigned dose (none of these patients required any additional transfusion or hemostatic adjunct after the intervention).

**Figure.**
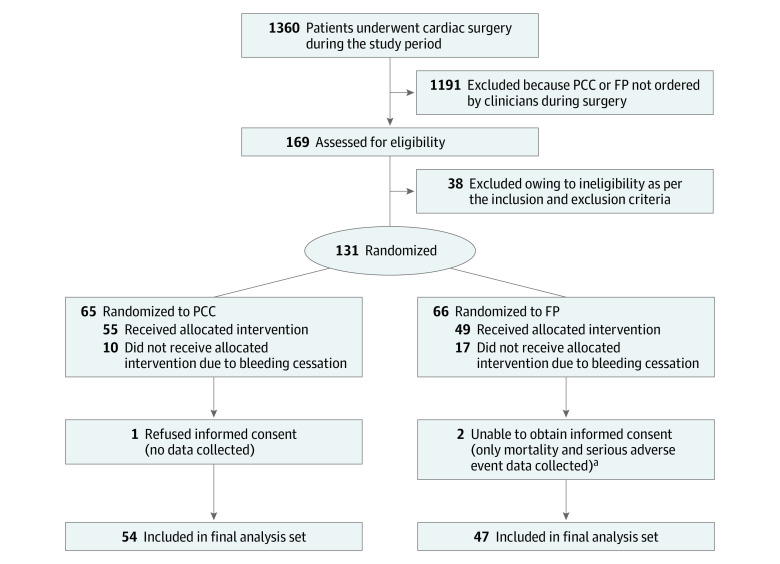
Enrollment, Randomization, and Treatment of Study Population FP indicates frozen plasma; PCC, prothrombin complex concentrate. ^a^Permission granted a priori by the research ethics boards.

The median age of the study population was 64 years (interquartile range [IQR], 54-73 years), 28 patients (28%) were female, and 82 patients (81%) underwent complex operations. Baseline demographic and surgical characteristics were well balanced (Table 1). The median time from end of cardiopulmonary bypass to administration of the first dose of the investigational product was similar in both groups (1.0 hour [IQR, 0.5-1.5 hours] in the PCC group vs 1.2 hours [IQR, 0.8-1.5 hours] in the FP group; *P* = .19) (Table 2). Patients in the PCC group received a median of 24.9 IU/kg (IQR, 21.8-27.0 IU/kg) of PCC, with 5 patients (9.3%) receiving 2 doses of PCC (Table 2) and 2 patients (3.7%; 95% CI, 0.4%-12.7%) receiving FP (10 U total) after having received 1 dose (1 patient) or 2 doses (1 patient) of PCC. Patients in the FP group received a median of 12.5 mL/kg (IQR, 10.0-15.0 mL/kg) of FP (assuming 1 U FP = 250 mL), with 9 patients (19.1%) receiving 2 doses of FP (Table 2). Four patients (8.5%; 95% CI, 2.4%-20.4%) received additional FP (20 U total) after receiving 2 doses of FP per the protocol, and none received any PCC. There were no notable between-group differences in perioperative laboratory values (Table 2).

**Table 1.** Demographic and Clinical Characteristics of the Study Population at Baseline

Characteristic	No. (%) of patients	
	PCC group (n = 54)	FP group (n = 47)
Age, median (IQR), y	66 (50-73)	67 (55-74)
Sex		
Female	14 (25.9)	14 (29.8)
Male	40 (74.1)	33 (70.2)
Race/ethnicity		
American Indian or Alaska Native	0	1 (2.1)
Asian or Pacific Islander	12 (22.2)	10 (21.3)
Black or African American	1 (1.8)	0
White	31 (57.4)	26 (55.3)
Other^a^	10 (18.5)	10 (21.3)
Weight, mean (SD), kg	81.0 (17.0)	79.3 (17.5)
BMI, mean (SD)	23.6 (4.5)	23.1 (4.7)
NYHA class^b^		
I (least severe)	14 (25.9)	11 (23.4)
II	22 (40.7)	19 (40.4)
III	15 (27.8)	15 (31.9)
IV (most severe)	3 (5.6)	2 (4.3)
Myocardial infarction		
None	48 (88.9)	42 (89.4)
0-90 d	2 (3.7)	3 (6.4)
>90 d	3 (5.6)	1 (2.1)
Ejection fraction, %		
>50	42 (77.8)	35 (74.5)
31-50	11 (20.4)	10 (21.3)
21-30	1 (1.8)	2 (4.3)
<21	0	0
Pulmonary pressure, mm Hg		
Unknown	6 (11.1)	6 (12.8)
<30	38 (70.4)	34 (72.3)
31-55	9 (16.7)	5 (10.6)
>55	1 (1.8)	2 (4.3)
Hypertension	36 (66.7)	31 (66.0)
Dyslipidemia	34 (63.0)	28 (59.6)
Congestive heart failure	12 (22.2)	13 (27.7)
Atrial fibrillation	8 (14.8)	7 (14.9)
Diabetes	11 (20.4)	10 (21.3)
Chronic lung disease	5 (9.3)	6 (12.8)
Stroke or TIA	5 (9.3)	7 (14.9)
Peripheral vascular disease	2 (3.7)	3 (6.4)
Active endocarditis	5 (9.3)	7 (14.9)
Preoperative dialysis	2 (3.7)	0
Preoperative laboratory values		
Creatinine, mg/dL		
No.	51	45
Median (IQR)	1.1 (0.8-1.4)	1.0 (0.9-1.2)
Hemoglobin, g/dL		
No.	52	46
Mean (SD)	13.2 (1.9)	12.8 (2.1)
Platelet count, ×10^3^/µL		
No.	52	46
Mean (SD)	205 (58)	210 (67)
International normalized ratio		
No.	52	44
Mean (SD)	1.2 (0.3)	1.2 (0.3)
Surgical factors		
Previous cardiac surgery	19 (35.2)	11 (23.4)
Nonelective surgery	11 (20.4)	15 (31.9)
Complex surgery^c^	45 (83.3)	37 (78.7)
Procedure, No. (% of procedures)		
Coronary artery bypass grafting	20 (37.0)	15 (31.9)
Aortic valve procedure	34 (63.0)	27 (57.4)
Mitral valve procedure	17 (31.5)	16 (34.0)
Tricuspid valve procedure	3 (5.6)	3 (6.4)
Pulmonary valve procedure	7 (13.0)	1 (2.1)
Surgery on aorta	18 (34.0)	24 (51.1)
Complex congenital	6 (11.1)	3 (6.4)
Other^d^	21 (38.9)	13 (27.6)
Cardiopulmonary bypass duration, mean (SD), min	172 (71)	166 (46)
Tranexamic acid dose, g		
No.	52	47
Mean (SD)	4.3 (2.3)	4.1 (1.3)
Heparin dose, mean (SD), IU	53 167 (18 107)	55 543 (20 996)
Protamine dose, mg		
No.	48	42
Mean (SD)	406 (98)	451 (151)
Cell salvage blood, mL		
No.	40	33
Mean (SD)	516 (660)	563 (554)

Abbreviations: BMI, body mass index (calculated as weight in kilograms divided by height in meters squared); FP, frozen plasma; IQR, interquartile range; NYHA, New York Heart Association; PCC, prothrombin complex concentrate; TIA, transient ischemic attack.

SI conversion factors: To convert creatinine to micromoles per liter, multiply by 88.4; hemoglobin to grams per liter, by 10.0; and platelet count to ×10^9^/L, by 1.0.

^a^ Other races marked as unknown or not applicable.

^b^ New York Heart Association functional classification: (I) No limitation of physical activity and no symptoms. (II) Slight limitation of physical activity; ordinary physical activity results in fatigue, palpitation, or dyspnea. (III) Marked limitation of physical activity; less than ordinary activity causes fatigue, palpitation, or dyspnea. (IV) Unable to carry on any physical activity without discomfort. Symptoms of heart failure at rest.

^c^ Procedures other than coronary artery bypass graft only, single valve only, or repair of atrial septal defect only.

^d^ Examples of other procedures include myectomy, atrial septal defect repair, left ventricular aneurysmectomy, and insertion of intra-aortic balloon pump.

**Table 2.** Details of Intervention, Laboratory Values, Bleeding Severity, and Hemostatic Therapies

Variable	PCC group (n = 54)	FP group (n = 47)	*P* value
Intervention details			
Dosage of investigational product			
Mean (SD)	25.9 (8.7) IU/kg	12.5 (7.4) mL/kg	Not applicable
Median (IQR)	24.9 (21.8-27.0) IU/kg	12.5 (10.0-15.0) mL/kg	
Doses of investigational product, No. (%)			.25^a^
1	49 (90.7)	38 (80.9)	
2	5 (9.3)	9 (19.1)	
Time from start of surgery to order of first dose, median (IQR), h	5.2 (4.2-5.9)	5.0 (4.3-5.4)	.29^b^
Time from end of CPB to administration of first dose, median (IQR), h	1.0 (0.5-1.5)	1.2 (0.8-1.5)	.19^b^
Time from administration of first dose to leaving the operating room, h^c^			.83^b^
No.	51	43	
Median (IQR)	1.1 (0.7-1.6)	1.2 (0.7-1.9)	
Laboratory values			
Hemoglobin, g/dL			
Intraoperative nadir			.18^d^
No.	54	47	
Mean (SD)	8.3 (1.4)	7.9 (1.3)	
Intraoperative post-CPB			.20^d^
No.	54	46	
Mean (SD)	10.0 (1.3)	9.7 (1.3)	
Day of surgery (last recorded value)			<.001^d^
No.	53	47	
Mean (SD)	10.7 (1.6)	9.5 (1.3)	
Postoperative day 1 (last recorded value)			.33^d^
No.	53	47	
Mean (SD)	9.4 (1.4)	9.1 (1.2)	
Platelet count, ×10^3^/µL			
Intraoperative post-CPB			.29^d^
No.	53	43	
Mean (SD)	116 (36)	125 (43)	
Day of surgery (last recorded value)			.09^d^
No.	52	47	
Mean (SD)	168 (51)	152 (42)	
Postoperative day 1 (last recorded value)			.63^d^
No.	53	47	
Mean (SD)	153 (56)	148 (52)	
International normalized ratio			
Intraoperative, post-CPB			.83^d^
No.	53	44	
Mean (SD)	2.3 (1.4)	2.2 (0.9)	
Day of surgery (last recorded value)			.11^d^
No.	50	46	
Mean (SD)	1.3 (0.3)	1.4 (0.2)	
Postoperative day 1 (last recorded value)			.81^d^
No.	53	46	
Mean (SD)	1.2 (0.3)	1.3 (0.2)	
Fibrinogen, mg/dL			
Intraoperative post-CPB			.36^d^
No.	51	45	
Mean (SD)	200 (60)	210 (70)	
Bleeding severity and hemostatic therapies			
Postintervention hemostatic therapy from 60 min to 4 h, No. (%)^e^			
No	43 (79.6)	32 (68.1)	.25^a^
Yes	11 (20.4)	15 (31.9)	
Postintervention hemostatic therapy from 60 min to 24 h, No. (%)^e^			
No	41 (75.9)	31 (66.0)	.28^a^
Yes	13 (24.1)	16 (34.0)	
Bleeding categories according to modified UDPB classification^f^			
Moderate (class 2), No. (%)	42 (79.2)	29 (61.7)	.08^a^
Severe or massive (classes 3 and 4), No. (%)	11 (20.8)	18 (38.3)	
Chest tube drainage, mL			
12 h			<.001^b^
No.	53		
Median (IQR)	310 (250-455)	500 (310-750)	
24 h			<.001^b^
No.	53		
Median (IQR)	450 (370-630)	700 (470-950)	
Reexploration, No. (%)	3 (5.6)	5 (10.6)	.47^a^
Fibrinogen concentrate, No. (%)	23 (42.6)	22 (46.8)	.69^a^
Recombinant factor VII, No. (%)	1 (1.9)	3 (6.4)	.34^a^

Abbreviations: CPB, cardiopulmonary bypass; FP, frozen plasma; IQR, interquartile range; PCC, prothrombin complex concentrate; UDPB, universal definition of perioperative bleeding.

SI conversion factors: To convert hemoglobin to grams per liter, multiply by 10.0; platelet count to ×10^9^/L, by 1.0; and fibrinogen to grams per liter, by 0.01.

^a^ Based on results of the Fisher exact test.

^b^ Based on results of the Wilcoxon signed rank test.

^c^ Excluding patients who were randomized in the operating room but received interventional product after leaving the operating room.

^d^ Based on results of the *t* test.

^e^ Includes receipt of any allogeneic blood transfusions, hemostatic adjuncts, or procedures from 60 minutes to 4 hours and from 60 minutes to 24 hours after intervention initiation.

^f^ Determinants used in this study included postoperative chest tube output; units of red blood cells, plasma, and platelets transfused; use of factor concentrates; and surgical reexploration.^21^ For this study the following components of the UDPB score were not used: delay in chest closure and use of cryoprecipitate. Because all patients received PCC or FP, none are classified as lower than 2. Data missing from 1 patient who died in the PCC group.

Bleeding severity and hemostatic therapy outcomes were all numerically lower in the PCC group. Hemostatic therapy was not required at the 4-hour time point for 43 patients (80%) in the PCC group and for 32 patients (68%) in the FP group (*P* = .25) nor at the 24-hour time point for 41 patients (76%) in the PCC group and for 31 patients (66%) patients in the FP group (*P* = .28) (Table 2). Individual and cumulative allogeneic blood component transfusions were also numerically lower in the PCC group for other explored time intervals (Table 3). For the 24-hour interval after start of surgery, the mean ratio of cumulative allogeneic blood components transfused was 42% lower (95% CI, 23%-55%; *P* < .001) in the PCC group, primarily because of the FP component, which was 94% lower (95% CI, 89%-97%; *P* < .001), followed by red cell transfusions, which was 31% lower (95% CI, 1%-53%; *P* = .05). After exclusion of FP administered as part of the investigational medicinal product, the median numbers of units were 6.0 U (IQR, 4.0-11.0 U) in the PCC group and 10.0 U (IQR, 6.0-16.0 U) in the FP group (ratio, 0.80; 95% CI, 0.59-1.08; *P* = .15) (Table 3). During the first 24 hours after start of surgery, 15 patients (28%) in the PCC group and 8 patients (17%) in the FP group received no red blood cells (*P* = .24). Chest tube drainage was significantly lower in the PCC group both at 12 hours (median volume, 310 mL [IQR, 250-455 mL] vs 500 mL [IQR, 310-750 mL]; *P* < .001) and at 24 hours (median volume, 450 mL [IQR, 370-630 mL] vs 700 mL [IQR, 470-950 mL]; *P* < .001) after surgery (Table 2).

**Table 3.** Allogeneic Blood Component Transfusions

Variable	PCC group			FP group			PCC:FP ratio (2-sided 95% CI) of LS mean	*P* value
	No. of patients	Median (IQR), U	LS mean (CI), U	No. of patients	Median (IQR), U	LS mean (CI), U		
Cumulative allogeneic blood components within 24 h after start of surgery^a^								
RBC + platelet + FP (including IMP)	54	6.0 (4.0-11.0)	8.7 (7.2-10.5)	47	14.0 (8.0-20.0)	14.8 (12.3-17.9)	0.58 (0.45-0.77)	<.001
RBC + platelet + FP (excluding IMP)	54	6.0 (4.0-11.0)	8.6 (7.0-10.6)	47	10.0 (6.0-16.0)	10.8 (8.6-13.4)	0.80 (0.59-1.08)	.15
Individual allogeneic blood components within 24 h after start of surgery								
FP (including IMP)	54	0	0.3 (0.2-0.4)	47	4.0 (3.0-4.0)	4.4 (3.6-5.3)	0.06 (0.03-0.11)	<.001
Platelet	54	4.0 (4.0-8.0)	6.2 (5.1-7.6)	47	8.0 (4.0-12.0)	7.2 (5.9-8.9)	0.86 (0.64-1.15)	.30
Red blood cell	54	1.5 (0.0-4.0)	2.2 (1.7-2.9)	47	3.0 (1.0-5.0)	3.2 (2.5-4.2)	0.69 (0.47-0.99)	.05
Cumulative allogeneic blood components within 24 h after CPB^b^								
RBC + platelet + FP (including IMP)	54	5.0 (4.0-9.0)	7.5 (6.2-9.1)	47	12.0 (7.0-19.0)	13.4 (11.1-16.2)	0.56 (0.43-0.73)	<.001
RBC + platelet + FP	54	5.0 (4.0-9.0)	7.4 (6.0-9.2)	47	8.0 (4.0-15.0)	9.5 (7.6-11.8)	0.78 (0.58-1.06)	.11
Individual allogeneic blood components within 24 h after CPB								
FP (including IMP)	54	0	0.3 (0.2-0.4)	47	4.0 (2.0-4.0)	4.2 (3.4-5.1)	0.06 (0.03-0.11)	<.001
Platelet	54	4.0 (4.0-8.0)	5.9 (4.8-7.3)	47	8.0 (4.0-12.0)	6.7 (5.4-8.4)	0.88 (0.65-1.19)	.40
Red blood cell	54	1.0 (0.0-2.0)	1.3 (1.0-1.8)	47	2.0 (1.0-3.0)	2.5 (1.9-3.2)	0.53 (0.36-0.78)	.001
Cumulative allogeneic blood components within 7 d after CPB								
RBC + platelet + FP (including IMP)	54	7.0 (5.0-11.0)	8.5 (7.1-10.1)	47	13.0 (8.0-19.0)	14.2 (11.9-16.9)	0.60 (0.47-0.76)	<.001
RBC + platelet + FP (excluding IMP)	54	7.0 (5.0-11.0)	8.4 (6.9-10.2)	47	10.0 (5.0-15.0)	10.3 (8.4-12.6)	0.81 (0.62-1.07)	.15
Individual allogeneic blood components within 7 d after CPB								
FP (including IMP)	54	0	0.3 (0.2-0.4)	47	4.0 (2.0-4.0)	4.2 (3.4-5.1)	0.06 (0.03-0.11)	<.001
Platelet	54	4.0 (4.0-8.0)	6.1 (4.9-7.5)	47	8.0 (4.0-12.0)	6.8 (5.5-8.5)	0.89 (0.66-1.21)	.50
Red blood cell	54	2.0 (1.0-3.0)	2.1 (1.7-2.7)	47	3.0 (1.0-4.0)	3.2 (2.6-4.0)	0.67 (0.48-0.92)	.01
Cumulative allogeneic blood components within 24 h after start of intervention								
RBC + platelet + FP (including IMP)	54	2.0 (0.0-5.0)	4.1 (3.1-5.6)	47	6.0 (4.0-14.0)	9.9 (7.3-3.4)	0.42 (0.27-0.64)	<.001
RBC + platelet + FP (excluding IMP)	54	2.0 (0.0-5.0)	4.1 (2.8-6.0)	47	2.0 (1.0-10.0)	5.8 (3.9-8.8)	0.70 (0.40-1.22)	.20
Individual allogeneic blood components within 24 h after start of intervention								
FP (including IMP)	54	0	0.3 (0.2-0.5)	47	4.0 (3.0-4.0)	4.4 (3.6-5.3)	0.06 (0.03-0.11)	<.001
Platelet	54	0.0 (0.0-4.0)	3.0 (1.8-4.9)	47	0.0 (0.0-8.0)	3.6 (2.1-6.1)	0.83 (0.40-1.72)	.60
Red blood cell	54	0.0 (0.0-1.0)	0.9 (0.6-1.3)	47	2.0 (0.0-3.0)	2.0 (1.4-2.7)	0.46 (0.28-0.76)	.003

Abbreviations: CPB, cardiopulmonary bypass; FP, frozen plasma; IMP, investigational medicinal product; IQR, interquartile range; LS, least-squares; PCC, prothrombin complex concentrate; RBC, red blood cell.

^a^ Units of allogenic blood components counted as follows: each RBC unit, 1 U; each 250 mL plasma unit, 1 U; and each platelet dose, 4 U.

^b^ If patients required repeated CPB, the end time of the last procedure was used for calculations.

Treatment emergent adverse events and thromboembolic events were similar between groups, as were the durations of mechanical ventilation, intensive care unit stay, and hospital stay (Table 4). No transfusion reactions were noted in either group. There were 2 deaths (3.7%) in the PCC group and 2 deaths (4.1%) in the FP group.

**Table 4.** Adverse Events and Other Measured Outcomes at 28-Day Follow-up

Outcome	No. (%) of patients [No. of events]	
	PCC group (n = 54)	FP group (n = 47)
Any adverse event^a^	42 (77.8) [108]	41 (87.2) [102]
Any serious adverse event^a^	19 (35.2) [29]	14 (28.6) [22]^b^
No.		49
Thromboembolic adverse events^a^	4 (7.4) [4]	4 (8.2) [5]^b^
No.		49
Stroke or TIA	2	3
Atrial thrombosis	0	1
Vascular thrombosis	2	1
Acute kidney injury, No. (%)^c^	4 (7.4)	3 (6.1)
Duration of mechanical ventilation, median (IQR), d	0.5 (0.4-0.9)	0.6 (0.4-0.9)
Duration of intensive care unit stay, median (IQR), d	2.0 (1.0-4.8)	3.0 (1.1-4.8)
Duration of hospitalization, median (IQR), d^d^	9.3 (8.0-13.7)	12.3 (9.2-14.5)

Abbreviations: FP, frozen plasma; IQR, interquartile range; PCC, prothrombin complex concentrate; TIA, transient ischemic attack.

^a^ Patients who experienced more than 1 event are counted only once in the totals.

^b^ Includes data on 2 patients for whom informed consent could not be obtained but research ethics board approval was obtained to collect serious adverse events.

^c^ Acute kidney injury was defined as greater than 2-fold increase in creatinine or kidney failure requiring hemodialysis within 28 days of surgery.

^d^ Censored at 28 days.

## Discussion

This study suggests that, for patients who experience excessive bleeding during cardiac surgery and require coagulation factor replacement as part of routine clinical care, PCC may be a suitable substitute for FP—only 2 patients who were randomized to PCC required FP in addition to PCC, albeit with a wide 95% CI of 0.4% to 12.7% around the 3.7% point estimate. Primarily owing to the avoidance of FP transfusions, the PCC group had 42% lower exposure (95% CI, 23%-55%; *P* < .001) to allogeneic blood components (Table 3), without any indication of increased risk of adverse events (Table 4). Study results also suggest that PCC may have hemostatic superiority over FP because, in addition to reduced FP transfusions, the PCC group had less blood loss (Table 2) and required fewer red cell transfusions (Table 3) than the FP group. Because this was a pilot study that was not powered to detect differences in the primary efficacy outcomes, these findings should be considered exploratory.

To date, several observational studies and a single randomized pilot study have compared the use of PCC with FP in cardiac surgery.^13,23^ Although the observational studies are heterogeneous, meta-analyses of their results suggest that PCC is associated with decreased blood loss and red cell transfusions,^13,23^ which is consistent with the findings of our study. The meta-analyses also explored the safety of PCC and found no association with thromboembolic adverse events, but did note a trend toward increased risk of kidney dysfunction.^13,23^ In our study, adverse event rates, including kidney dysfunction, were similar between the study groups. The 1 randomized clinical trial comparing the use of PCC and FP in cardiac surgery was a single-center study that included 50 patients and was conducted to determine the recruitment rate for a larger study and to assess safety (no issues were identified).^24^

Although PCCs do not contain the full complement of procoagulant factors that are present in FP, they have several important characteristics that may confer hemostatic superiority over FP. First, transfusion of PCC may lead to greater increases than FP in procoagulant to anticoagulant levels (eg, factor II to antithrombin), thereby enhancing thrombin generation.^25^ Second, unlike the highly variable coagulation factor content of FP,^26^ PCCs are standardized according to their factor IX content, which may lead to more reliable factor replenishment and response.^12^ Third, PCCs are more concentrated than FP; thus, a substantially lower PCC volume is required to achieve dose equivalence for increasing thrombin generation (a standard dose of 25 IU/kg PCC is 80 mL, whereas a standard dose of 15 mL/kg FP is 1000 mL).^15^ Thus, PCCs are less likely than FP to exacerbate the obligatory hemodilution that occurs during cardiac surgery, which is an important cause of anemia and red cell transfusion.^27,28^

Despite the reduced blood loss and red cell transfusions in the PCC group, suggesting hemostatic superiority to FP, exposure to other blood components, namely platelets and fibrinogen concentrate, was similar. This discordance is most likely due to the stepwise approach to coagulation management at the participating institutions.^20^ Recognizing that the most affected parameters in cardiac surgery are platelet count and function and fibrinogen levels,^2^ the recommended order of therapy was to first administer platelets and fibrinogen concentrate (cryoprecipitate was not used), followed by FP or PCC to increase coagulation factor levels and thrombin generation.

One objective of this study was to assess the feasibility of the study protocol, which was met. We successfully identified the approximately 12% of patients who were deemed to require coagulation factor replacement by the surgical team and appropriately randomized patients who were eligible for the study. The postoperative consent process was also successful because consent could not be obtained for only 3 patients. Important aspects of the protocol were adhered to in both groups, with the sole exception being the between-group difference (FP > PCC) in the number of patients who received less than 80% of the assigned dose. This difference can be attributed to the usual practice of titrating blood component administration to treatment effect. Because a full dose of FP (10-15 mL/kg) represents a substantially larger volume and takes longer to administer than PCC, clinicians would have had a longer opportunity to observe an incremental treatment effect during FP administration, leading them to not administer the full recommended dose. The doses used in this study reflected existing guideline recommendations^29^ and were consistent with the doses used for approved indications^30^ as well as institutional practice. Patients who did not receive the full dose of FP were not disadvantaged because none of them required additional transfusions or hemostatic adjuncts.

### Limitations

One limitation of the study is that, because it compared the products under usual conditions of care, a standardized transfusion protocol could not be strictly enforced, which may have led to practice variability. Both hospitals, however, had 1 established transfusion algorithm, and processes of care measures (such as timing of therapy, nadir hemoglobin levels, and other coagulation parameters) were similar between the groups, suggesting that the transfusion practice was consistent between groups. Another limitation is that, owing to the nature of the therapeutics, treating clinicians could not be blinded to group assignment (although such blinding was maintained until the decision was made to initiate treatment). Finally, as already noted, this was a pilot study, and between-group comparisons were exploratory.

## Conclusions

For patients who require coagulation factor replacement for bleeding during cardiac surgery, this pilot study illustrates that a multicenter randomized trial comparing PCC with FP is feasible. Our results suggest that PCC may be a suitable substitute for FP because it markedly decreases the need for FP and may have hemostatic superiority without increasing the occurrence of adverse events. Adequately powered multicenter randomized clinical trials are warranted to delineate the risk-benefit profile of PCC relative to FP for the management of bleeding during cardiac surgery and other settings.

## Notes

Supplement 1. **Trial Protocol**

Supplement 2. **Data Sharing Statement**
