# Supplementary material for: Comparison of 4-Factor Prothrombin Complex Concentrate With Frozen Plasma for Management of Hemorrhage During and After Cardiac Surgery: A Randomized Pilot Trial
Source: JAMA Netw Open. 2021 Apr 1;4(4):e213936. doi: 10.1001/jamanetworkopen.2021.3936 (PMC8017469; doi:10.1001/jamanetworkopen.2021.3936)
Supplement: Supplement 1. — Trial Protocol [file jamanetwopen-e213936-s001.pdf]

CLINICAL STUDY PROTOCOL

FARES

FActor REplacement in Surgery

Prothrombin complex concentrate versus frozen plasma in bleeding adult cardiac surgical patients

**Investigational Product:**	*Octaplex*
**Indication:**	Bleeding cardiac surgery patients requiring coagulation factor replacement
**Study Design:**	Multicentre, randomized, active-control, pragmatic Phase 2 pilot study
**Sponsor:**	Keyvan Karkouti
**Study Number:**	FARES
**EudraCT and/or IND Number:**	
**Development Phase:**	Phase 2
**Planned Clinical Start:**	Quarter 3 2019
**Planned Clinical End:**	Quarter 2 2020
**Date of Protocol:**	13-June-2019
**Version:**	1.0
**Coordinating Investigator:**	**Keyvan Karkouti MD**<br>Department of Anesthesia and Pain Management<br>Toronto General Hospital<br>200 Elizabeth Street, 3EN<br>Toronto, ON<br>M5G 2C4<br>Canada

STUDY OUTLINE

**Name of Sponsor:**<br>Keyvan Karkouti, Toronto General Hospital	
**Name of Investigational Product:**<br>*Octaplex*	**Protocol Identification Code:**<br>FARES
**Name of Active Ingredient:**<br>Prothrombin complex concentrate	**Date of Final Protocol:**

Title of Study:

Prothrombin complex concentrate versus frozen plasma in bleeding adult cardiac surgical patients: A multicentre, randomized, active-control, pragmatic, Phase 2 pilot study

Indication:

Bleeding cardiac surgery patients requiring coagulation factor replacement

Study Centres:

Toronto General Hospital (University Health Network), and Sunnybrook Health Sciences Centre, Toronto, Canada

Objectives:

To inform the design and primary outcome parameter of a definitive Phase 3 trial comparing the efficacy and safety of 4-factor prothrombin complex concentrate (PCC; *Octaplex)* versus frozen plasma (FP) in bleeding cardiac surgical patients in whom coagulation factor replacement with PCC or FP is ordered according to accepted clinical standards.

Study Design:

This is a multicentre, randomized, active-control, pragmatic, Phase 2 pilot study in adult cardiac surgery patients. Two Canadian hospitals will participate, and it is estimated that the study will take approximately 9 months to complete.

Approximately 120 bleeding adult cardiac surgical patients who require coagulation factor replacement during cardiac surgery will be included. Patients will be randomized to receive either PCC or FP when the blood bank receives the first order for coagulation factor replacement and deems it to be in accordance with accepted clinical standards. Patients will be treated according to their assigned group on the first and second times when coagulation factor replacement is ordered during the treatment period (24 hours after randomization). For any additional doses (i.e., the third dose and thereafter), patients in both groups will receive FP (in 1U increments at the discretion of the ordering physician). No other aspects of care will be modified.

This pilot study aims to select a clinically relevant primary efficacy endpoint for a confirmative Phase 3 study, which will subsequently aim to determine if PCC is non-inferior or superior to FP in terms of efficacy

Name of Sponsor:<br>Keyvan Karkouti, Toronto General Hospital	
**Name of Investigational Product:**<br>*Octaplex*	**Protocol Identification Code:**<br>FARES
**Name of Active Ingredient:**<br>Prothrombin complex concentrate	**Date of Final Protocol:**

and safety in bleeding cardiac surgical patients. In the pilot study, safety outcomes will be measured for the first 28 days, which is the duration of participation of each patient in the trial.

Number of Patients:

A total of 100 evaluable patients, with fifty evaluable patients per arm. Approximately 120 patients will need to be randomized to compensate for randomized but untreated patients.

Patient Selection Criteria:

Inclusion Criteria:

Patients undergoing any index cardiac surgery with or without CPB in whom coagulation factor replacement with PCC or FP is ordered in the operating room for:

1. Management of bleeding, or
2. Anticipated bleeding in a patient who has
   a) been on-pump for >2 hours, or
   b) undergone a complex procedure (e.g., aortocoronary bypass [ACB] plus aortic valve replacement).

Coagulation factor deficiency must either be known to exist (as indicated by elevated EXTEM clotting time [CT] or international normalized ratio [INR]), or be suspected based on the clinical situation.

Exclusion Criteria:

Patients who meet any of the following criteria are *not* eligible for the study:

1. Undergoing heart transplantation, insertion or removal of ventricular assist devices (not including intra-aortic balloon pump [IABP]), or repair of thoracoabdominal aneurysm
2. Critical state immediately before emergency surgery with high probability of death within 24 hours of surgery (e.g., acute aortic dissection, cardiac arrest within 24 hours before start of surgery)
3. History of heparin induced thrombocytopenia
4. Last preoperative INR >1.5 and patient on warfarin
5. Taken dabigatran, rivaroxaban, apixaban, or edoxaban within 48 hours of start of surgery
6. Administered PCC or FP within 48 hours before start of surgery
7. History of severe allergic reaction to PCC or FP
8. Refusal of allogeneic blood products due to religious or other reasons
9. Known pregnancy

**Name of Sponsor:**<br>Keyvan Karkouti, Toronto General Hospital	
**Name of Investigational Product:**<br>*Octaplex*	**Protocol Identification Code:**<br>FARES
**Name of Active Ingredient:**<br>Prothrombin complex concentrate	**Date of Final Protocol:**

Test Product, Dose, and Mode of Administration:

Octaplex and FP will be administered intravenously. Each dose of *Octaplex* will be 1500 IU for patients with IBW≤60 kg or 2000 IU for patients with IBW>60 kg (corresponding to weight-base dosing range of 20–25 IU/kg_{IBW} rounded up to nearest 500 IU increment, up to a maximum of 2000 IU per dose). Each dose of FP will be 3 U for patients with IBW≤60 kg or 4 U for patients with IBW>60 kg (corresponding to weight-base dosing range of 10–15 mL/kg_{IBW} rounded up to 1 U increment, up to a maximum of 4 U per dose).

If an order for a second dose is received, the second dose of *Octaplex* or FP will be released and administered to patients according to the randomized group allocation as per the first dose. For any additional doses (i.e., the third dose and thereafter), patients in both groups will receive FP (in 1 U increments at the discretion of the ordering physician).

Duration of Treatment:

Twenty-four hours from initial randomization (which occurs in the operating room). Thereafter, patients will be treated as per standard practices.

Study Outcome Parameters (Endpoints):

This pilot study aims to select a clinically relevant primary efficacy endpoint for a confirmative Phase 3 study.

Efficacy Endpoints:

Primary Endpoints:

1. Treatment response, defined as effective if no additional hemostatic intervention (such as administration of hemostatic agents including a second dose of IMP, platelet transfusion, or surgical re-exploration) from 60 minutes to 4 and 24 hours after initiation of the first dose of IMP (otherwise will be defined as not effective). If the dose of IMP is repeated within less than 60 minutes, treatment response to the IMP will be assessed for the 60 minutes after the second IMP dose only.
2. Amount of allogeneic blood products (for each type of product and cumulatively) administered during the first 24 hours after the start of surgery.
3. Number of patients who do not receive any RBC transfusions or any allogeneic blood transfusions during the first 24 hours after the start of surgery.

Name of Sponsor: Keyvan Karkouti, Toronto General Hospital	
**Name of Investigational Product:** *Octaplex*	**Protocol Identification Code:** FARES
**Name of Active Ingredient:** Prothrombin complex concentrate	**Date of Final Protocol:**

Exploratory Endpoints:

1. Number of partial or full IMP doses of PCC and FP administered during the first 24 hours after the start of surgery.
2. Incidence of major bleeding, using the validated e-CABG (1) and universal definition of perioperative bleeding (UDPB) in cardiac surgery (2) scores and their individual components during the first 24 hours after start of surgery, as well as a modified version of the UDPB not including PCC and FP requirements.
3. Amount of allogeneic blood products (for each type of product and cumulatively) from start of surgery to postoperative day (POD)-7.
4. Number of patients who do not receive any RBC transfusions or any allogeneic blood transfusions from start of surgery to POD-7.
5. Number of patients receiving recombinant activated factor VII (rFVIIa) during the first 24 hours after the start of surgery
6. Number of patients receiving fibrinogen concentrate during the first 24 hours after the start of surgery.
7. Change in coagulation parameters (INR, ROTEM [EXTEM CT and MCF] and fibrinogen levels) within 75 minutes before and after IMP administration, where performed as part of standard of care.
8. Time elapsed from first IMP administration to time leaving the operating room.

Safety Endpoints:

All adverse events (AEs) and serious AEs (SAEs) will be collected from beginning of surgery (defined as entry into OR) to POD-28.

1. SAEs will be collected individually and as a composite (death, myocardial infarction, stroke, acute liver injury, acute kidney injury and thromboembolic events).
2. Incidence of transfusion-related acute lung injury (TRALI) and transfusion-associated circulatory overload (TACO) as per the International Society of Blood Transfusion (ISBT) (3) imputability of ‘possible’ or higher will also be collected.

**Name of Sponsor:**<br>Keyvan Karkouti, Toronto General Hospital	
**Name of Investigational Product:**<br>*Octaplex*	**Protocol Identification Code:**<br>FARES
**Name of Active Ingredient:**<br>Prothrombin complex concentrate	**Date of Final Protocol:**

Additional Endpoints:

1. Duration of mechanical ventilation (measured as duration of ventilation and ventilator-free days) up to POD-28.
2. Duration of intensive care unit (ICU) stay up to POD-28.
3. Duration of hospitalization up to POD-28.
4. Mortality up to POD-28.
5. Compliance of transfusion practice with the study's hemostatic algorithm (4), based on review of patient charts by 2 blinded adjudicators. This will include comparison of lowest hemoglobin concentration during the first 24 hours after the start of surgery (4).

Study Procedures:

When the first order for PCC or FP is received by the blood bank, the technologist will confirm patient eligibility with the clinical team. Following confirmation of eligibility, the technologist will randomize the patient to PCC or FP according to the randomization schedule and prepare and release the product in a tamper sealed container (with weight device in PCC boxes to ensure adequate concealment). The operating room personnel will be blinded to group allocation until the tamper lock seal is broken in the operating room immediately prior to infusion. The rate and route of administration of products will be documented according to current standards.

If a second order is received within 24 hours of randomization, the blood bank technologist will release IMP as determined by the randomization schedule, as per usual practice (a tamper-sealed container will not be used for the second order). For subsequent orders in the operating room and intensive care unit (ICU), the technologist will release FP for both groups.

The type of IMP administered will not be recorded in the chart to ensure that the research personnel assessing outcomes and AEs are blinded to assignment. Patients will also be blinded to treatment allocation.

Study Visits:

Visit 1: First post-randomization visit (0 to 24 hours after randomization)

Obtain consent from patient or surrogate
Collect baseline and surgical data
Collect laboratory, transfusion, and bleeding data
Collect extubation time
Collect concomitant medications
Collect AEs and SAEs

**Name of Sponsor:**<br>Keyvan Karkouti, Toronto General Hospital	
**Name of Investigational Product:**<br>*Octaplex*	**Protocol Identification Code:**<br>FARES
**Name of Active Ingredient:**<br>Prothrombin complex concentrate	**Date of Final Protocol:**

Visit 2: Postoperative days 2-7 (or at discharge if earlier)
Obtain consent from patient or surrogate (if not already done)
Collect laboratory and transfusion data
Collect extubation time, length of stay in the ICU and hospital (if applicable)
Collect AEs and SAEs

Visit 3: Postoperative day 28 (in person if in hospital or by phone)
Obtain consent from patient or surrogate (if not already done)
Collect AEs and SAEs
Collect extubation time, length of stay in the ICU, length of stay in the hospital, 28-day mortality

Concomitant Therapies and Management:

Participating hospitals will use a standardized point-of-care based transfusion algorithm that will employ a targeted approach to correction of coagulopathy (as per the validated Transfusion Avoidance in Cardiac Surgery [TACS] algorithm) (4), but with selection of PCC or FP determined according to the patient's assigned grouping and using the doses described above. Recommended RBC transfusion thresholds will be 75 g/L during surgery and 85 g/L in bleeding or unstable patients.

Ethics:

This is a pragmatic trial that compares two therapies that are currently within the standard of care for this procedure and poses no additional risks to patients and entails no additional interventions or laboratory testing outside of normal clinical care. Moreover, due to the emergency nature of the condition being studied (i.e., bleeding during or after surgery), the trial will include only patients who are incapable of providing informed consent at the time the therapy is needed and in whom delays in obtaining surrogate consent can be severely detrimental to their well-being. Thus, the study meets the criteria of the Tri-council policy statement for the ethical conduct for research involving humans for alteration to consent requirement. We will obtain patient or surrogate consent at the earliest possible opportunity after surgery from patients or their surrogate.

Sample Size and Statistical Analysis Plan:

The study will include 120 randomized patients, which, accounting for randomized but untreated patients in both arms, is expected to provide at least 100 evaluable patients, with 50 in each arm. The sample size determination was based on pragmatic reasons, no statistical sample size estimation was performed.

In general, descriptive statistics with 95% exploratory confidence intervals will be used to describe the data. Simple tests of comparison will be used to compare outcomes between the two groups. To detect trends of

Name of Sponsor:<br>Keyvan Karkouti, Toronto General Hospital	
**Name of Investigational Product:**<br>*Octaplex*	**Protocol Identification Code:**<br>FARES
**Name of Active Ingredient:**<br>Prothrombin complex concentrate	**Date of Final Protocol:**

a different efficacy or safety pattern, the relationship of efficacy and safety endpoints with procedural or patient characteristics will be explored. For this, descriptive statistics within subgroups or model-based exploratory testing and confidence intervals will be computed.

FLOW CHART OF ASSESSMENTS

Table 1 Flow Chart of Assessments Performed Throughout the Study

Procedures	Prior to enrolment	Visit 1 Post-randomization (0 to 24 h)*	Visit 2 POD 2-7/DC	Visit 3 POD-28
**Blood bank receives PCC or FP order[a]**	X			
**Inclusion and exclusion criteria**	X			
**Randomization**	X			
**IMP administration[b]**		X		
**Patient (SDM) debriefing and consent**		X	(X)	(X)
**Baseline data**				
Demographics		X		
Medical history		X		
Preoperative medications		X		
**Surgical data**				
Intraoperative medications		X		
CPB time		X		
Cross-clamp time		X		
Circulatory arrest		X		
Fluid in- and output monitoring		X		
Inotropes and vasopressors		X		
**Laboratory assessments**				
Chemistry[c]		X	X	
Hematology[c]		X	X	
Coagulation profile[c d]		X[d]	X	
Safety labs[c]		X	X	
**Transfusion requirements**				
RBCs		X	X	
Pooled and apheresis platelets		X	X	
Plasma		X	X	
Fibrinogen concentrates		X	X	
Cryoprecipitate		X	X	
Other hemostatic products		X	X	
**Blood loss determination (e-CABG, UDPB and modified UDPB[e])**		X	X	
**Extubation time**		X	(X)	(X)
**ICU length of stay**		X	(X)	(X)
**Hospital length of stay**			X	(X)
**AEs and SAEs**		X	X	X
**Concomitant medications**		X	X	X

AE, adverse event; CPB, cardiopulmonary bypass; DC, discharge; ICU, intensive care unit; POD, postoperative day; SAE, serious adverse event; UDPB, universal definition of perioperative bleeding; e-CABG, European Coronary Artery Bypass Grafting SDM- Surrogate Decision Maker

*For any activities not completed during this visit, additional visits will be undertaken to complete activities

[a] After the start of surgery.

[b] IMP will be administered during surgery based on the physician's judgement.

[c] As per standard practice

[d] Within 75 minutes before and after IMP administration

[e] Modified version of the UDPB not including PCC and FP requirements.

( ) If needed

PROTOCOL SIGNATURES

This study is intended to be conducted in compliance with the protocol, Good Clinical Practice and applicable regulatory requirements.

Keyvan Karkouti MD [signature] 2019/07/24

Coordinating Investigator and Sponsor	Signature	Date

Department of Anesthesia
Toronto General Hospital
200 Elizabeth Street, 3EN
Toronto, ON

Hans-Peter Hucke [signature] 2019-07-24

Statistician	Signature	Date

ERGOMED CDS mbH
Im Mediapark 2
D-50670 Cologne
Germany

TABLE OF CONTENTS

STUDY OUTLINE **2**
FLOW CHART OF ASSESSMENTS **9**
PROTOCOL SIGNATURES **10**
TABLE OF CONTENTS **11**
LIST OF ABBREVIATIONS **14**
1 INTRODUCTION **16**
1.1 BACKGROUND 16
1.2 RATIONALE FOR CONDUCTING THE STUDY 19
1.3 BENEFIT-RISK STATEMENT 19
1.4 PRINCIPAL INVESTIGATOR (SPONSOR) 20
2 STUDY OBJECTIVES **21**
3 INVESTIGATIONAL PLAN **22**
3.1 ENDPOINTS 22
3.1.1 Efficacy Endpoints *22*
3.1.2 Safety Endpoints *23*
3.1.3 Additional Endpoints *23*
3.2 OVERALL STUDY DESIGN AND PLAN 23
3.3 DISCUSSION OF STUDY DESIGN AND CHOICE OF CONTROL GROUP 24
3.3.1 Study Aims *24*
3.3.2 Dose Rationale *24*
3.3.3 Choice of Comparator *25*
3.3.4 External Validity *25*
3.3.5 Randomization and Baseline Differences *25*
3.3.6 Recruitment and Informed Consent *25*
3.3.7 Blinding of Investigational Medicinal Product *26*
3.3.8 Drop-outs and Crossovers *26*
3.3.9 Outcome Assessments and Independent Data and Safety Monitoring Committee (IDSMC) *26*
4 STUDY POPULATION **27**
4.1 POPULATION BASE 27
4.1.1 Inclusion Criteria *27*
4.1.2 Exclusion Criteria *27*
4.2 PRIOR AND CONCOMITANT THERAPY 27
4.2.1 Permitted Concomitant Therapy *27*
4.2.2 Forbidden Concomitant Therapy *28*
4.3 WITHDRAWAL AND REPLACEMENT OF PATIENTS 28
4.3.1 Premature Patient Withdrawal *28*
4.3.2 Patient Replacement Policy *28*
4.4 ASSIGNMENT OF PATIENTS TO TREATMENT GROUPS 28
4.5 RELEVANT PROTOCOL DEVIATIONS 28
4.6 SUBSEQUENT THERAPY 28

5 INVESTIGATIONAL MEDICINAL PRODUCTS ... 29
5.1 CHARACTERIZATION OF INVESTIGATIONAL PRODUCTS ... 29
5.1.1 Octaplex ... 29
5.1.2 Frozen Plasma ... 30
5.2 BREAKING THE STUDY BLIND ... 31
5.3 TREATMENT COMPLIANCE ... 31
5.3.1 IMP Dispensing and Accountability ... 31
5.3.2 Assessment of Treatment Compliance ... 31
6 STUDY CONDUCT ... 32
6.1 STUDY PROCEDURE ... 34
6.1.1 Prior to Enrolment ... 34
6.1.2 Visit 1: First post-randomization visit (0 to 24 hours after randomization) ... 34
6.1.3 Visit 2: Postoperative days 2-7 (or at discharge if earlier) ... 35
6.1.4 Visit 3: Postoperative day 28 (in person if in hospital or by phone) ... 35
6.1.5 Time Windows Used in this Study, including Tolerances ... 35
6.2 DURATION OF STUDY ... 36
6.2.1 Planned Duration for an Individual Patient ... 36
6.2.2 Planned Duration for the Study as a Whole ... 36
6.2.3 Premature Termination of the Study ... 36
7 ASSESSMENTS AND METHODS ... 37
7.1 BASELINE DATA ... 37
7.1.1 Demographic and Baseline Characteristics ... 37
7.1.2 Medical History and Prior/Concomitant Medications ... 37
7.2 STUDY ASSESSMENTS ... 37
7.2.1 Surgical and Surgery-Related Data ... 37
7.2.2 Transfusion Data ... 37
7.2.3 Bleeding Data ... 37
7.3 LABORATORY ASSESSMENTS ... 38
7.3.1 Test Parameters and Laboratories (Only if performed as part of standard of care) 38
7.3.2 Blood Sampling ... 39
7.3.3 Citrated and Fresh Blood ... 39
7.3.4 Serum ... 39
7.3.5 Recording of Clinically Significant Abnormal Laboratory Values as AEs/ADRs 39
7.4 SAFETY ASSESSMENTS ... 39
7.4.1 Assessments for Safety Endpoints ... 39
7.4.2 Adverse Events (AEs) ... 40
7.4.3 Serious Adverse Events (SAEs) ... 42
7.4.4 SAE Reporting Timelines ... 43
7.4.5 Other Relevant Safety Information ... 43
8 DATA HANDLING AND RECORD KEEPING ... 45
8.1 DOCUMENTATION OF DATA ... 45
8.1.1 Source Data and Records ... 45
8.1.2 Case Report Forms ... 45
8.1.3 Data Validation Procedures ... 45
8.2 INFORMATION TO INVESTIGATORS ... 46
8.3 RESPONSIBILITIES ... 46

8.4 Investigator's Site File 46
8.5 Provision of Additional Information 47
8.6 Independent Data Safety Monitoring Committee 47

9 STATISTICAL METHODS AND SAMPLE SIZE 48
9.1 Determination of Sample Size 48
9.2 Statistical Analysis 48
9.2.1 Safety Analysis Plan 49
9.2.2 Subgroup Analysis 50
9.2.3 Handling of Missing Data 50
9.3 Randomization, Stratification, and Code Release 50

10 ETHICAL/REGULATORY, LEGAL AND ADMINISTRATIVE ASPECTS 51
10.1 Ethical/Regulatory Framework 51
10.2 Approval of Study Documents 51
10.3 Consent Issues 51
10.4 Protocol Amendments 52
10.5 Confidentiality of Patient Data 52

11 QUALITY CONTROL AND QUALITY ASSURANCE 53
11.1 Periodic Monitoring 53
11.2 Audit and Inspection 53

12 REPORTING AND PUBLICATION 54
12.1 Clinical Study Report 54
12.2 Publication Policy 54

13 LIABILITIES AND INSURANCE 55

14 REFERENCES 56

15 APPENDICES 61
15.1 AE and SAE Flow Chart for Assessment and Reporting 61
15.2 Consent and Data Collection Guidelines at TGH 62

LIST OF ABBREVIATIONS

Abbreviation	Description
ACB	Aortocoronary Bypass
ADR	Adverse Drug Reaction
AE	Adverse Event
ALT	Alanine Aminotransferase
AST	Aspartate Aminotransferase
BMI	Body Mass Index
CI	Confidence Interval
CPB	Cardiopulmonary Bypass
CRF	Case Report Form
CRO	Contract Research Organization
E-CABG	European Coronary Artery Bypass Grafting
eCRF	Electronic Case Report Form
EDC	Electronic Data Capture
EXTEM	ROTEM Assay Assessing the Extrinsic Coagulation Pathway
GCP	Good Clinical Practice
HIV	Human Immunodeficiency Virus
IABP	Intra-Aortic Balloon Pump
ICU	Intensive Care Unit
IDSMC	Independent Data Safety Monitoring Committee
IMP	Investigational Medicinal Product
INR	International Normalized Ratio
ITT	Intention-To-Treat
MCF	Maximum Clot Firmness
IV	Intravenous
MedDRA	Medical Dictionary for Regulatory Activities
OR	Operating Room
PCC	Prothrombin Complex Concentrate
POD	Postoperative Day
PP	Per-Protocol
PRV	Pseudorabies Virus
PT	Prothrombin Time
PTT	Partial Thromboplastin Time
RBC	Red Blood Cell
REB	Research Ethics Board
rFVIIa	Recombinant Activated Factor VII
SAE	Serious Adverse Event
SAF	Safety Analysis Population

**Abbreviation**	**Description**
SBV	Schmallenberg Virus
SDM	Surrogate Decision Maker
SDV	Source Data Verification
TACS	Transfusion Avoidance in Cardiac Surgery
TEAE	Treatment Emergent Adverse Event
TEE	Thromboembolic Event
UDPB	Universal Definition of Perioperative Bleeding
WFI	Water for Injections

1 INTRODUCTION

1.1 Background

Bleeding in cardiac surgery

Cardiac surgery is frequently complicated by coagulopathic bleeding that often leads to excessive blood loss, blood product transfusion and bleeding-related complications (5-8). Bleeding and transfusions are associated with increased morbidity and mortality, including up to an 8-fold increase in the odds of death (6), and patients with major bleeding are at particularly high risk of adverse outcomes (e.g., infection, heart failure, and mortality) (9-21).

Coagulopathy during cardiac surgery is caused by several factors, including the contact of blood with the cardiopulmonary bypass (CBP) circuit, which activates the intrinsic and extrinsic coagulation pathways (despite the use of heparin and heparin-coated circuits), causing excessive clot formation and breakdown. Other contributory factors include hemodilution, hypothermia, blood loss, surgical trauma, and use of foreign substances such as aortic grafts causing the consumption of coagulation factors (22-26). These in turn can lead to excessive fibrinolysis and fibrinogen deficiency, platelet dysfunction and thrombocytopenia (24, 25). Deficiency of enzymatic coagulation factors (e.g., vitamin K-dependent factors II, VII, IX, X, and XI) is also an important cause of coagulopathy, leading to impaired generation of thrombin, which is considered to be central in the coagulation cascade (27, 28). Activated thrombin converts soluble fibrinogen into insoluble strands of fibrin, which forms the basis of the clot, and it catalyzes numerous other coagulation-related reactions (29); consequently, replacement of coagulation factors is an important aspect of a multimodal approach to coagulopathy to reduce bleeding and transfusion (8, 30).

To replenish depleted coagulation factors and improve thrombin generation, two therapeutics, frozen plasma (FP) and prothrombin complex concentrate (PCC), are available. FP is currently the mainstay of therapy for patients with acquired coagulopathies in North America whereas PCC is the mainstay of therapy in much of Europe (31).

Frozen Plasma

FP is obtained from donated whole blood and contains all enzymatic coagulation factors, although some coagulation factor activity is lost during storage and processing (including freezing for storage and thawing prior to transfusion) (32). FP utilized in North America is not pathogen-reduced. FP is the mainstay of therapy for bleeding cardiac surgery patients requiring coagulation factor replacement in many countries, and is administered in around 15% of all cardiac surgeries in the United States (33). Despite being a mainstay of therapy, there is no clinical trial evidence for the use of FP in bleeding cardiac surgery patients requiring coagulation factor replacement. A systematic review investigating the use of FP identified only one study that tested the interventional (i.e., not prophylactic) use of FP in patients undergoing cardiac surgery (34). In this study, two types of FP were compared but a randomized control group was not included, making it difficult to draw conclusions about the efficacy and safety of FP (35). When also considering trials that tested FP prophylactically, the authors concluded that there is no evidence for the efficacy of FP in the prevention of bleeding for patients undergoing cardiac surgery but some evidence for an increase in RBC transfusion in those treated with FP. Moreover, in a prospective cohort study of 967 patients undergoing cardiac surgery, FP transfusion was not associated with improved 30-day mortality rates (36).

The lack of evidence for the efficacy of FP is concerning in the context of the risks associated with its use (37, 38). FP can lead to adverse events (AEs) including allergic reactions in 1–3% of transfusions, and while most are not serious, life-threatening anaphylaxis can occur (34). Transfusion-related acute lung injury (TRALI) is associated with plasma-containing components and is a leading cause of transfusion-related death (34, 39). Also, transfusion-associated circulatory overload (TACO), which occurs as a result of the large volume of transfusion required to achieve therapeutic effect with FP, occurs in approximately 5% of transfusions and is fatal in 2% (40). FP transfusion is associated with transmission of infectious diseases, as FP is not usually filtered or treated with solvent/detergent (41). These adverse effects are often dose dependent (42-45). FP requires ABO blood group compatibility matching and thawing, which can delay therapy. The large volumes of FP needed to effectively raise thrombin generation can further delay time to hemostatic control and lead to substantial hemodilution, resulting in additional RBC transfusions (46).

Prothrombin Complex Concentrate

PCC offers a potential alternative to FP for treating bleeding cardiac surgery patients requiring coagulation factor replacement. PCCs contain prothrombin and other enzymatic coagulation factors, the anticoagulant proteins C and S and anti-thrombin, and small amounts of heparin; they are routinely defined as 3-factor (that contain factors II, IX, and X) or 4-factor (that contain factors II, VII, IX, and X) formulations (47). PCCs are purified from human pooled plasma, which is fractionated into cryoprecipitate and cryoprecipitate-free plasma fractions through a process of slow thawing, then eluted from cryoprecipitate-free plasma (47). The production of PCCs includes strict viral inactivation using solvents, detergents, pasteurization, nanofiltration, and vapor-heated treatment (5, 47).

PCCs have several potential advantages over FP. Solvent/detergent treatment and filtering to remove viruses substantially reduces the risk of transmission of infectious agents with PCCs. Indeed, a Consensus Conference recommended in 2007 that pathogen reduction technologies should be implemented to improve the safety of transfusion when they became available (48). Unlike FP, PCCs do not require ABO compatibility matching or thawing, and can therefore be prepared and administered more quickly. PCCs are associated with a substantially lower risk of TRALI (due to pooling of the source donor plasma), and also a lower risk of TACO (37), as substantially lower volumes of PCC are required than with FP to achieve dose-equivalence for increasing thrombin generation (for example, in a 70-kg patient, a standard dose of 25 IU/kg PCC would be administered in a volume of 80 mL, whereas a standard dose of 15 mL/kg FP would be administered in a volume of 1000 mL) (46). PCCs contain standardized levels of coagulation factors and thus have a more predictable therapeutic effect than FP, whose coagulation factor concentrations vary depending on the characteristics of the donor (49, 50).

PCCs do not contain the full balanced complement of procoagulants and anticoagulants present in FP (51) and it is therefore conceivable that they might be less effective, although *in vitro* studies suggest that PCCs may be more effective than FP in enhancing thrombin generation after cardiac surgery (46). Also, because PCCs contain more procoagulants than anticoagulants, they may carry a higher risk of thrombotic events, disseminated intravascular coagulation and acute kidney injury (47, 52-56). Finally, since PCCs contain heparin, they are contraindicated in patients who are allergic to heparin or have a prior history of heparin induced thrombocytopenia.

Review of the literature

Few guidelines exist on the use of PCC in acquired coagulopathies. The European Society of Anaesthesiology recommends goal-directed therapy with coagulation factor concentrates, including PCC, since this may reduce transfusions during cardiac surgery (57), and the American Society of Anesthesiologists suggests PCCs for excessive bleeding in the presence of elevated INR (58). However, there is little high-quality clinical evidence to support these recommendations: there are few direct comparisons of PCC versus FP for managing coagulopathy resulting from coagulation factor deficiency during cardiac surgery (or other types of surgery) and randomized controlled trials have not yet been performed.

We carried out an observational study that compared outcomes for patients at Toronto General Hospital who received FP (*n*=1151), PCC (*n*=79), or both (*n*=125) for management of coagulopathy after cardiac surgery between 2012 and 2016 (59). Using propensity score matching, we matched 117 patients who received FP with 117 patients with similar risk and surgery profiles who received PCC (with or without FP). (As part of the Risk Factor Validation study [REB#: 16-5649]. The odds ratio (OR) for red cell transfusion avoidance was 2.4-fold (95% confidence interval [CI] 1.2–4.8) higher for patients receiving PCC. In addition, incidences of massive transfusion (OR 0.58; 95% CI 0.33–1.0) and refractory bleeding (OR 0.49; 95% CI 0.24–1.03) tended to be lower in patients who received PCC. Results from this exploratory study suggest that PCC is potentially more efficacious than FP in avoiding transfusion, and AE profiles were similar between groups, suggesting a comparable safety profile.

Our results are similar to those from other studies. A recent systematic review only identified 4 small, non-randomized studies – including our own (59) – comparing perioperative PCCs to FP in cardiac surgery (60). The meta-analysis showed PCCs were associated with reduced risk of RBC transfusion (OR 2.22; 95%CI 1.45-3.40) and fewer units of RBCs transfused (OR 1.34; 95%CI 0.78-1.90). PCCs were not associated with increased hospital mortality (OR 0.94; 95%CI 0.59-1.49), stroke (OR 0.80; 95%CI 0.41-1.56) or AKI occurrence (OR 0.80; 95%CI 0.58-1.12), suggesting that PCCs are more effective than FP without decreasing safety. In one of the studies in the systematic review, Arnekian and colleagues (61) compared three groups of cardiac surgical patients: 24 who received only PCC, 26 who received only FP, and 27 who received both products. In an unadjusted analysis, they found that RBC transfusions occurred least frequently in the PCC group and most frequently in patients who received both products. However, given the small sample size and lack of risk-adjusted analysis, few conclusions can be drawn from this study. In another study that included only patients who underwent pulmonary endarterectomy with cardiopulmonary bypass (CPB), Ortmann and colleagues (62) compared transfusion requirements and outcomes between 55 patients who received only FP and 45 patients who received only PCC for management of post-CPB coagulopathic bleeding. In an unadjusted analysis, they found that the PCC group had lower blood loss than the FP group, but transfusion rates were similar. They conducted multivariable regression analysis for AEs and found no differences between the two groups. However, given its small sample size, limited generalizability, and lack of risk-adjustment for efficacy outcomes, this study should be interpreted with caution. In a larger study, Cappabianca and colleagues (63) used propensity scores to match 225 patients who received PCC (with or without FP) to 225 patients who received only FP for management of post-CPB coagulopathy. They found that RBC transfusion requirements were lower in the PCC group (84% versus 93%), as were multiple (more than two units) RBC transfusions (51% versus 70%) and number of units of RBCs transfused (3.4 versus 5.2 units). While there were no major between-group differences in adverse outcomes, in a regression analysis that incorporated the propensity score the authors noted an association between PCC use and acute kidney injury that led them to caution about this potential risk. Similarly, in a recent multi-centre observational study not included in the above systematic

review, Biancari and colleagues compared 101 patients who received FP following coronary artery bypass grafting to 101 propensity-score-matched patients who received PCC with or without FP (64). PCC was associated with a significant decrease in rate of RBC transfusions (67.3% vs. 83.2%), but was also associated with an increased risk of acute kidney injury. However, there were many limitations to this study, including unknown differences in PCC dosage, bleeding severity and coagulopathy. Furthermore, in the group of patients that received both FP and PCC, it was not known what the order of administration was and so PCC could have been given as salvage therapy (64). Together, these studies suggest that PCC may be more efficacious than FP in cardiac surgery; however, data from well-designed, prospective, randomized trials are lacking.

1.2 Rationale for Conducting the Study

The proposed pilot study will determine the feasibility, and inform the design and primary outcome parameter, of a definitive Phase 3 trial comparing the efficacy and safety of the PCC *Octaplex* versus FP in bleeding cardiac surgical patients in whom coagulation factor replacement with PCC or FP is ordered according to accepted clinical standards. In addition, the results from the pilot study will be used in sample size calculations for the full study and safety data will be compared to detect any early safety issues. A better homogeneity of the study cohort can be expected if only a single PCC, rather than any registered PCC, is admitted for comparison.

The primary objective of the potential future Phase 3 study will be to determine if PCC is non-inferior or superior to FP in terms of efficacy and safety in consecutive bleeding cardiac surgical patients in whom coagulation factor replacement (with PCC or FP) is ordered according to accepted clinical standards. By using a randomized controlled design and multiple centres to increase generalizability and statistical power to detect differences in adverse events, the confirmative Phase 3 study will address many of the limitations of previous studies.

Hemostatic management in bleeding surgical patients is evolving from empirical therapy with non-purified allogeneic blood products to targeted therapy with purified products that have undergone treatment with pathogen reduction technologies (65). The proposed study, by comparing two currently available but distinctly different therapies for treating bleeding surgical patients requiring coagulation factors, is well aligned with this change. Given the potential advantages of PCC over FP detailed above (viral inactivation, ease of administration, standardized dosing/predictable effect, and substantially lower risk of TRALI and TACO due to smaller volume), we believe that a finding of superiority or non-inferiority will lead to the use of PCC in place of FP for the management of bleeding surgical patients requiring coagulation factor replacement.

1.3 Benefit-Risk Statement

Substituting either PCC or FP for the other is not expected to pose any material risks to the participants. Patients will only be included in the trial when their clinicians have ordered coagulation factor replacement for treatment of bleeding, according to accepted clinical standards. The two coagulation factor replacement therapies, *Octaplex* (the PCC to be used in this study) and FP, are currently within the standard of care for this procedure. *Octaplex* is currently approved for use in Canada for the "treatment of bleeding and perioperative prophylaxis of bleeding in acquired deficiency of prothrombin complex coagulation factors, such as deficiency caused by treatment with vitamin K antagonists, or in case of overdose of vitamin K antagonists, when rapid correction of the deficiency is required" (66). Neither *Octaplex* nor FP pose additional risks to patients and

entail no additional interventions outside of normal clinical care. No patient will receive coagulation factor replacement solely for the purposes of this study.

The experience to date with this PCC has shown an excellent safety profile (67-71). As discussed, PCC is pathogen reduced, can be administered quickly and in predictable doses, and has a substantially lower risk of TRALI and TACO compared with FP, making its administration likely to be both safer than, and at least as efficacious as, FP. It is possible that treatment with PCC may not be as effective as FP given that it does not contain the full complement of clotting factors that plasma contains. By limiting maximal PCC dosing to well within acceptable limits that have been proven safe in other settings (67-71), we are confident that the potential risk of thrombotic complications has been addressed.

1.4 Principal Investigator (Sponsor)

The Sponsor and Coordinating Investigator of this study is Keyvan Karkouti MD at the Department of Anesthesia and Pain Management, Toronto General Hospital, 200 Elizabeth Street, 3EN, Toronto, ON, Canada.

Octapharma AG will support the conduct of this study by awarding an unrestricted grant for study conduct, supporting data management and statistical services.

2 STUDY OBJECTIVES

The main objective of this pilot trial is to inform the design and primary outcome parameter for a definitive Phase 3 trial comparing the efficacy and safety of PCC (*Octaplex)* versus FP in bleeding cardiac surgical patients in whom coagulation factor replacement with PCC or FP is ordered according to accepted clinical standards.

3 INVESTIGATIONAL PLAN

3.1 Endpoints

This pilot study aims to select a clinically relevant primary efficacy endpoint for a confirmative Phase 3 study.

3.1.1 Efficacy Endpoints

Primary Endpoints:

1. Treatment response, defined as effective if no additional hemostatic intervention (such as administration of hemostatic agents including a second dose of IMP, platelet transfusion, or surgical re-exploration) from 60 minutes to 4 and 24 hours after initiation of the first dose of IMP (otherwise will be defined as not effective). If the dose of IMP is repeated within less than 60 minutes, treatment response to the IMP will be assessed for the 60 minutes after the second IMP dose only.
2. Amount of allogeneic blood products (for each type of product and cumulatively) administered during the first 24 hours after the start of surgery.
3. Number of patients who do not receive any RBC transfusions or any allogeneic blood transfusions during the first 24 hours after the start of surgery.

Exploratory Endpoints:

1. Number of partial or full IMP doses of PCC and FP administered during the first 24 hours after the start of surgery.
2. Incidence of major bleeding, using the validated e-CABG and universal definition of perioperative bleeding (UDPB) in cardiac surgery (1, 72) scores and their individual components during the first 24 hours after start of surgery, as well as a modified version of the UDPB not including PCC and FP requirements.
3. Amount of allogeneic blood products (for each type of product and cumulatively) from start of surgery to postoperative day (POD)-7.
4. Number of patients who do not receive any RBC transfusions or any allogeneic blood transfusions from start of surgery to POD-7.
5. Number of patients receiving recombinant activated factor VII (rFVIIa) during the first 24 hours after the start of surgery
6. Number of patients receiving fibrinogen concentrate during the first 24 hours after the start of surgery.
7. Change in coagulation parameters (INR, ROTEM [EXTEM CT and MCF] and fibrinogen levels) within 75 minutes before and after IMP administration, where performed as part of standard of care.
8. Time elapsed from first IMP administration to time leaving the operating room.

3.1.2 Safety Endpoints

All AEs and SAEs will be collected from beginning of surgery (defined as entry into OR) to POD-28.

SAEs will be collected individually and as a composite (death, myocardial infarction, stroke, acute liver injury, acute kidney injury and thromboembolic events).

Incidence of all transfusion reactions, including transfusion-associated circulatory overload (TACO) and transfusion-related acute lung injury (TRALI) as per the International Society of Blood Transfusion (ISBT) (16) imputability of 'possible' or higher will be compared.

3.1.3 Additional Endpoints

The following additional endpoints will be collected:

1. Duration of mechanical ventilation (measured as duration of ventilation and ventilator-free days) up to POD-28.
2. Duration of intensive care unit (ICU) stay up to POD-28.
3. Duration of hospitalization up to POD-28.
4. Mortality up to POD-28.
5. Compliance of transfusion practice with the study's hemostatic algorithm based on review of patient charts by 2 blinded adjudicators. This will include comparison of lowest hemoglobin concentration during the first 24 hours after the start of surgery.

3.2 Overall Study Design and Plan

This is a multicentre, randomized, active-control, pragmatic, Phase 2 pilot study in adult cardiac surgery patients. Two Canadian hospitals (Toronto General and Sunnybrook hospitals, Toronto) will participate and it is estimated that the study will take approximately 9 months to complete.

Approximately 120 bleeding adult cardiac surgical patients who require coagulation factor replacement after cardiac surgery, according to accepted clinical standards, will be included. Patients will be randomized to receive equivalent doses of either PCC (*Octaplex*) or FP when the blood bank receives the first order for coagulation factor replacement and deems it to be in accordance with accepted clinical standards. Patients will be treated according to their assigned group on the first and second times when coagulation factor replacement is ordered during the treatment period (up to 24 hours after randomization) [see flowchart on the next page]. For any additional doses (i.e., the third dose and thereafter), patients in both groups will receive FP (in 1 U increments at the discretion of the ordering physician). No other aspects of care will be modified.

Figure 1. Study Flow

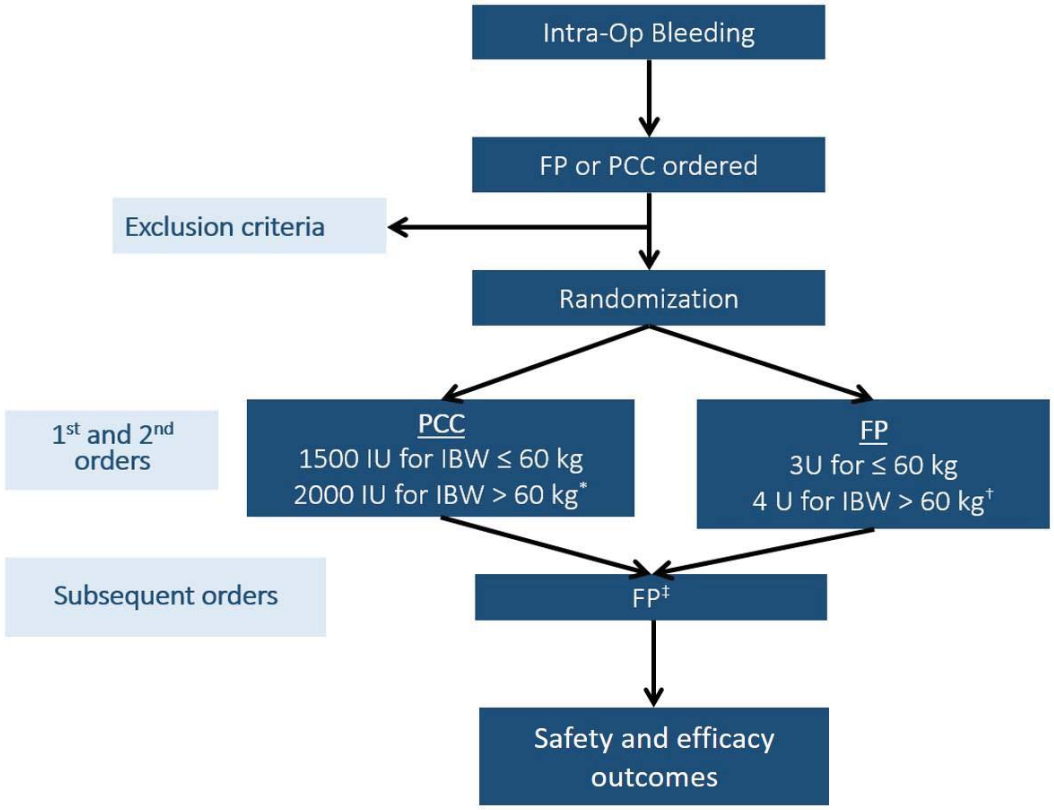

*PCC dose corresponds to a weight-based dosing range of 20–25 IU/kg_{IBW} rounded up to nearest 500 IU increment, up to a maximum of 2000 IU per dose

†FP dose corresponds to a weight-based dosing range of 10–15 mL/kg_{IBW} rounded up to the nearest 1 U increment, up to a maximum of 4 U per dose

‡FP in 1 U increments at the discretion of the ordering physician

PCC, prothrombin complex concentrate; FP, frozen plasma; IU, international units; RBC, red blood cell concentrate

3.3 Discussion of Study Design and Choice of Control Group

3.3.1 Study Aims

This pilot study aims to select a clinically relevant primary efficacy endpoint for a phase 3 study, which will subsequently aim to determine if PCC is non-inferior or superior to FP in terms of the selected primary efficacy and safety endpoints in bleeding cardiac surgical patients.

3.3.2 Dose Rationale

Each time coagulation factor replacement is ordered, patients will receive either *Octaplex* (1 dose = 1500 IU for IBW≤60 kg or 2000 IU for IBW>60 kg; based on 20–25 IU/kg_{IBW}, rounded up to nearest 500 IU increment

on a standard dosing chart, up to a maximum of 2000 IU per dose) or FP (1 dose = 3 U for IBW≤60 kg or 4 U for IBW>60 kg; based on 10–15 mL/kg_{IBW}, rounded up to the nearest 1 U increment, up to a maximum of 4 U for each order) according to their group assignment. If an order for a second dose is received, the second dose of *Octaplex* or FP will be released and administered to patients according to the randomized group allocation as per the first dose. For any additional doses (i.e., the third dose and thereafter), patients in both groups will receive FP (in 1 U increments at the discretion of the ordering physician).

The doses of PCC and FP selected for the study represent current clinical practice at the study sites (choice currently decided by the cardiac anesthesiologist) and will only be given to patients in whom the therapies are clinically indicated as per the ordering physicians. There is an absence of a consensus of guidelines regarding PCC dosing in acquired coagulation factor deficiency; however, the dose of PCC is consistent with current clinical practice and is similar to doses used previously in other studies in a cardiac setting (4, 31, 59, 73). It is also equal to or lower than doses of PCC that have demonstrated a good safety and tolerability profile in other clinical settings (e.g., factor Xa inhibitor reversal in major bleeding (68, 69, 74) and trauma (70, 75).

3.3.3 Choice of Comparator

The trial will not include a placebo arm because delaying coagulation factor replacement in bleeding patients may expose them to the negative consequences of excessive blood loss, is not consistent with standard practice (76, 77), and would withhold an effective treatment from patients and thus be unethical. Moreover, the question being addressed does not meet any of Freedman's five conditions that would justify the use of a placebo control, which are: 1) no standard treatment exists; 2) standard treatment is not better than placebo; 3) standard treatment is a placebo or no treatment; 4) new evidence has shown uncertainty of the risk-benefit profile of the standard treatment; and 5) effective treatment is not readily available due to cost or supply issues (77, 78).

3.3.4 External Validity

The definitive phase 3 study will be a pragmatic study performed in multiple hospitals with different characteristics. Moreover, patients will be recruited and randomized after the clinical team orders coagulation factor replacement and the only change to routine practice is dictating the choice of coagulation factor replacement amongst the two therapies that are currently available and used interchangeably. For these reasons, the phase 3 study will have good external validity.

3.3.5 Randomization and Baseline Differences

Given the size of this pilot study and random patient assignment stratified by centre, study groups should be reasonably balanced with respect to important clinical variables and this should allow selection of an appropriate endpoint for the confirmative Phase 3 study. The random allocation schedule will be prepared by a biostatistician not involved in the conduct of the trial, and neither the individual randomizing nor any of the health care providers will know which treatment will be assigned to the patient when coagulation factor replacement is ordered.

3.3.6 Recruitment and Informed Consent

This is a pragmatic trial that compares two coagulation factor replacement therapies that are currently within the standard of care for this procedure and poses no additional risks to patients and entails no additional interventions outside of normal clinical care. Moreover, due to the emergency nature of the condition being studied

(i.e., bleeding during or after surgery), the trial will include only patients who are incapable of providing informed consent at the time the therapy is needed and in whom delays in obtaining surrogate consent can be severely detrimental to their well-being. Thus, this study qualifies for alteration to consent requirement before randomization. However, we will obtain consent from patients or their surrogate after surgery. The alteration to the informed consent process meets the criteria of the Tri-council policy statement for the ethical conduct for research involving humans, as is outlined in **Section 10.3**.

3.3.7 Blinding of Investigational Medicinal Product

Given that the products have quite different physical properties, it is not possible to blind treating clinicians to group assignment. To minimize bias, treating clinicians will be blinded to group assignment until immediately prior to IMP infusion. The blood bank technologist will randomize the patient to PCC or FP, according to the randomization schedule, and prepare and release the product in a tamper sealed container (with weight device in PCC boxes to ensure adequate concealment). The operating room personnel will be blinded to group allocation until the tamper lock seal is broken in the operating room immediately prior to infusion. For the second order, a tamper-sealed container will not be used and the blood bank technologist will release IMP as determined by the randomization schedule, as per usual practice. The type of IMP administered will not be recorded in the chart to ensure that the research personnel assessing outcomes and AEs are blinded to assignment. Patients will also be blinded to treatment allocation.

3.3.8 Drop-outs and Crossovers

Drop-outs: We anticipate that <20% of randomized patients will either not receive the treatment, will not have undergone an index cardiac procedure, or will not consent to remain in the study. The majority will likely be the first reason, in whom coagulation factor replacement will be deemed to be not necessary after it was ordered but before it is administered due to cessation of bleeding or identification of other causes of bleeding. Study sample size has been calculated to compensate for drop-outs.

Product switching: Other than the coagulation factor replacement order being cancelled, all patients will be treated according to the randomization schedule on the first and second times when coagulation factor replacement is ordered for the treatment period (24 hours after randomization). Following this, all patients will receive FP when coagulation factor replacement is ordered. To ensure minimal product switching, instructions will be entered into the blood bank information system to dictate the randomization product for the 24 hours after randomization and will flag the laboratory technologists if attempts are made to override the instruction.

3.3.9 Outcome Assessments and Independent Data and Safety Monitoring Committee (IDSMC)

An IDSMC will review accumulating safety, endpoint, and other study data (recruitment, retention and compliance, data quality and timeliness, risk vs. benefit). The function of the IDSMC will be to protect and serve the recruited patients particularly pertaining to patient safety as well as to assist and advise the Sponsor on medical questions and issues of study conduct and continuation. The IDSMC will be independent of the investigating team and the Sponsor in operating and formulating recommendations. The IDSMC will review relevant data during the course of the study. The full role of the IDSMC will be detailed in the IDSMC Charter.

4 STUDY POPULATION

4.1 Population Base

The study will include 120 randomized bleeding adult cardiac surgical patients who require coagulation factor replacement. This should provide 100 evaluable patients, accounting for drop-outs in both arms, with approximately 50 patients assigned to each of the two treatment groups.

4.1.1 Inclusion Criteria

Patients undergoing any index cardiac surgery with or without CPB in whom coagulation factor replacement with PCC or FP is ordered in the operating room for:

1. Management of bleeding, or
2. Anticipated bleeding in a patient who has
   a) been on-pump for >2 hours, or
   b) undergone a complex procedure (e.g., aortocoronary bypass [ACB] plus aortic valve replacement).

Coagulation factor deficiency must either be known to exist (as indicated by elevated EXTEM clotting time [CT] or international normalized ratio [INR]) or be suspected based on the clinical situation.

4.1.2 Exclusion Criteria

Patients who meet any of the following criteria are *not* eligible for the study:

1. Undergoing heart transplantation, insertion or removal of ventricular assist devices (not including intra-aortic balloon pump [IABP]), or repair of thoracoabdominal aneurysm
2. Critical state immediately before emergency surgery with high probability of death within 24 hours of surgery (e.g., acute aortic dissection, cardiac arrest 24 hours before surgery)
3. History of heparin induced thrombocytopenia
4. Last preoperative international normalized ratio (INR) >1.5 and patient on warfarin
5. Taken dabigatran, rivaroxaban, apixaban, or edoxaban within 48 hours of start of surgery
6. Taken PCC or plasma within 48 hours before start of surgery
7. History of severe allergic reaction to PCC or plasma
8. Refusal of allogeneic blood products due to religious or other reasons
9. Known pregnancy.

4.2 Prior and Concomitant Therapy

Details on medications taken within 1 week before enrolment and any concomitant medications taken during the study must be recorded in the case report form (CRF).

4.2.1 Permitted Concomitant Therapy

Participating hospitals will use a standardized point-of-care based transfusion algorithm that will employ a targeted approach to correct coagulopathy (as per the validated TACS algorithm) (4), but with selection of PCC or FP determined according to the patient's assigned grouping and using the doses described in **Section**

3.3.2. Recommended RBC transfusion thresholds will be 75 g/L during surgery and 85 g/L in bleeding or unstable patients.

Concomitant administration of any therapies required as part of standard patient care is permitted but must be recorded in the CRFs. All hemostatic drugs or products administered (e.g., heparin and protamine dose, anti-fibrinolytic drugs, desmopressin, rFVIIa, idarucizumab, andexanet alpha, or topical hemostatic agents) will be recorded, as well as all procedures that may influence amount of bleeding (e.g., retrograde autologous priming of CPB circuit, cell salvage). In addition, concomitant medications used to treat SAEs will be reported throughout the duration of follow-up (up to POD-28).

4.2.2 Forbidden Concomitant Therapy

No concomitant therapies are forbidden, except for the use of PCCs other than *Octaplex* (unless unavailable in emergency situations).

4.3 Withdrawal and Replacement of Patients

4.3.1 Premature Patient Withdrawal

Patients have the right to withdraw from the study at any time for any reason, without the need to justify their decision. The Investigator also has the right to withdraw patients for reasons such as intervention no longer indicated due to stoppage of bleeding, randomization of ineligible patients, and adverse events attributed to previous doses of IMP. For any withdrawals after study entry, the Investigator will obtain all the required details and document the reason(s) for discontinuation, and report to the IDSMB and REB where indicated. If the reason for withdrawal of a patient is an AE, the main specific event or laboratory test will be recorded, and the Investigator will make thorough efforts to clearly document the outcome.

4.3.2 Patient Replacement Policy

Patients withdrawn from the study for safety reasons will not be replaced.

4.4 Assignment of Patients to Treatment Groups

Patients will be assigned to treatment with either PCC or FP using a permuted-block, stratified (by centre) random allocation scheme prepared by a biostatistician not involved in the conduct of the trial. Group allocation will apply to the first two doses of IMP during the treatment period (up to 24 hours from randomization). For any additional doses (i.e., the third dose and thereafter), patients in both groups will receive FP. Subjects/patients are not permitted to re-enroll in the study.

4.5 Relevant Protocol Deviations

In the case of any major protocol deviation, the Investigator (Sponsor) will decide on the further participation of the patient in this study.

4.6 Subsequent Therapy

Any subsequent hemostatic therapy will be according to institutional standard of care.

5 INVESTIGATIONAL MEDICINAL PRODUCTS

5.1 Characterization of Investigational Products

5.1.1 Octaplex

Octaplex is a product derived from human plasma containing the coagulation factors II, VII, IX, and X and proteins C and S. It is manufactured by chromatographic purification of cryoprecipitate-poor plasma.

The *Octaplex* manufacturing process includes two dedicated virus inactivation/removal steps, by way of a solvent/detergent viral inactivation process and a virus removal nanofiltration step. The solvent/detergent treatment causes enveloped viruses such as pseudorabies virus (PRV), Schmallenberg virus (SBV), and human immunodeficiency virus [HIV] type 1 to be irreversibly destroyed. Nanofiltration removes infectious agents from protein solutions on the basis of their size may be the only method to date permitting efficient removal of enveloped (e.g., HIV-1, SBV, PRV, bovine viral diarrhea virus) and non-enveloped viruses (e.g., hepatitis A virus) under conditions where 90–95% of protein activity is recovered (79). Other precautions against viral transmission include: selection of plasma donors, screening of donations and plasma pool, as well as quality control measurements of the final product.

Composition of Octaplex

Octaplex is a human PCC for intravenous (IV) use. Its ingredients are listed in **Table 2**.

Table 2 Composition of *Octaplex*

Ingredients	Quantity per 20-mL vial	Quantity per 40-mL vial
**Active substances**		
Human coagulation factor II	280–760 IU	560-1520 IU
Human coagulation factor VII	180–480 IU	360-960 IU
Human coagulation factor IX	500 IU	1000 IU
Human coagulation factor X	360–600 IU	720-1200 IU
**Further active ingredients**		
Protein C	260–620 IU	520–1240 IU
Protein S	240–640 IU	480–1280 IU
**Clinically relevant non-medicinal ingredients**		
Heparin	80–310 IU	160–620 IU
Sodium citrate	17.0–27.0 mmol/L	17.0–27.0 mmol/L

IU, international units

Further excipient: Solvent (Water for Injection)

Small amounts of the S/D reagents TNBP (5 µg/ml) and Polysorbate 80 (50 µg/mL) may remain in the finished product. These substances are added during the manufacturing process because of their capacity to inactivate lipid-enveloped viruses.

Conditions for Storage and Use

The IMP has to be stored at room temperature (not more than 25°C) and protected from light. The product must not be frozen. The Investigator/authorized personnel at the site will ensure that the IMP is stored in appropriate conditions with restricted access and in compliance with national regulations.

Dose and Dosing Schedule

Patients randomized to *Octaplex* will receive 1500 IU for IBW≤60 kg or 2000 IU for IBW>60 kg (corresponding to weight-base dosing range of 20–25 IU/kg_{IBW} rounded up to nearest 500 IU increment, up to a maximum of 2000 IU per dose) the first and second occasions when coagulation factor supplementation is ordered during the first 24 hours after randomization. If an order for a second dose is received, the second dose of *Octaplex* or FP will be released and administered to patients according to the randomized group allocation as per the first dose. For any additional doses (i.e., the third dose and thereafter), patients in both groups will receive FP (in 1 U increments at the discretion of the ordering physician).

Patient weight	Octaplex dose	Plasma dose
≤60 kg	1500 IU	3 U
>60 kg	2000 IU	4 U

Preparation

Each vial of *Octaplex* will be reconstituted with 20 mL or 40 mL WFI (as per the manufacturer's instructions) at room temperature (not more than 25°C). *Octaplex* dissolves at room temperature to an almost colorless and slightly opalescent solution within 10 minutes. It will not be used if it remains cloudy or contains particulates.

Method of Administration

Octaplex will be administered intravenously, immediately after reconstitution as recommended, via free-flowing IV syringe injection. *Octaplex* should not be mixed with other medicinal products or crystalloid intravenous solutions.

Packaging and Labeling

Commercial supplies of *Octaplex* will be used. Several batches of IMP may be used throughout the study. The batch numbers will be recorded in the CRFs and reported in the final study report.

5.1.2 Frozen Plasma

Patients randomized to FP will receive 3 U for IBW≤60 kg or 4 U for IBW>60 kg (corresponding to weight-base dosing range of 10–15 mL/kg_{IBW} rounded up to 1 U increment, up to a maximum of 4 U per dose) for each order during the first 24 hours after randomization. FP will be provided by the Canadian Blood Services and will be stored, thawed, and pooled by the blood bank according to current standards. FP will be infused as per standard hospital protocols at the participating institutions (i.e., infused using timing recommended in the current monograph through standard 140 μm blood infusing set).

5.2 Breaking the Study Blind

This is a partially blinded randomized study, with patients and outcome assessors blinded to treatment allocation. Given the physical differences in the products and the emergency nature of the intervention, attending clinicians will not be blinded to the treatment. Thus, breaking the study blind is not an issue in this study.

The random allocation schedule will be prepared by a biostatistician not involved in the conduct of the trial (see **Section 9.3**). To minimize bias, neither the individual randomizing nor any of the health care providers will know which treatment will be assigned to a given patient when coagulation factor replacement is ordered. Blood products will be transported to the OR in weighted temper-proof boxes to maintain the blind until IMP infusion.

The type of IMP administered will not be recorded in the chart to ensure that the research personnel assessing outcomes and AEs are blinded to assignment. Patients will also be blinded to treatment allocation.

5.3 Treatment Compliance

5.3.1 IMP Dispensing and Accountability

IMP dispensing and accountability procedures will be as per standard practice at the participating centres.

5.3.2 Assessment of Treatment Compliance

Coagulation factor replacement will be ordered and administered by the clinical team in the hospital and will not be dependent on patient compliance.

6 STUDY CONDUCT

All patients having cardiac surgery with or without CPB at the study centres will be the potential patients in the study. Patients will be randomized if they bleed or are thought to have a high potential to bleed (see section 4.1.1) after the start of surgery and the clinical team determines that coagulation factor replacement is required according to current clinical standards for up to 24 hours after randomization. Once the clinical team orders coagulation factor replacement, the blood bank technologist will confirm patient eligibility and randomize the patient (according to a prepared randomization schedule) to *Octaplex* or FP and prepare the product.

For 24 hours from randomization, patients may receive 1500 IU *Octaplex* for IBW≤60 kg or 2000 IU *Octaplex* for IBW>60 kg, or 3 U FP for IBW≤60 kg or 4 U FP for IBW>60 kg, as per randomized group allocation. If an order for a second dose is received, the second dose of *Octaplex* or FP will be released and administered to patients according to the randomized group allocation as per the first dose. For any additional doses (i.e., the third dose and thereafter), patients in both groups will receive FP (in 1 U increments at the discretion of the ordering physician).

Standard coagulation measures, including INR, EXTEM CT and MCF, and fibrinogen level, will be obtained as per usual clinical practice ideally within 75 minutes before and after each IMP administration. Due to the nature of bleeding in these cases, however, IMP doses can be administered before these results are obtained or the test results are released by the laboratory. Clinical indications for IMP administration are bleeding in the setting of suspected or confirmed low coagulation factor levels, as indicated by EXTEM CT >90 seconds, which is the treatment threshold recommended by the validated and standardized TACS algorithm (see Figure 2; next page) (4), or INR >1.5. In certain circumstances, such as uncontrolled bleeding, clinicians may deem it necessary to administer coagulation factor replacement in cases where EXTEM CT is <90 seconds (or INR is <1.5 if ROTEM not being used).

All AEs and SAEs occurring until POD-28 will be recorded. Patients will be followed by research coordinators in each institution. All clinical outcomes will be obtained from patients' medical records and electronic records, history and physical where needed, and via phone contact during the follow-up visits.

The flow chart of assessments by study visit is given on page 9.

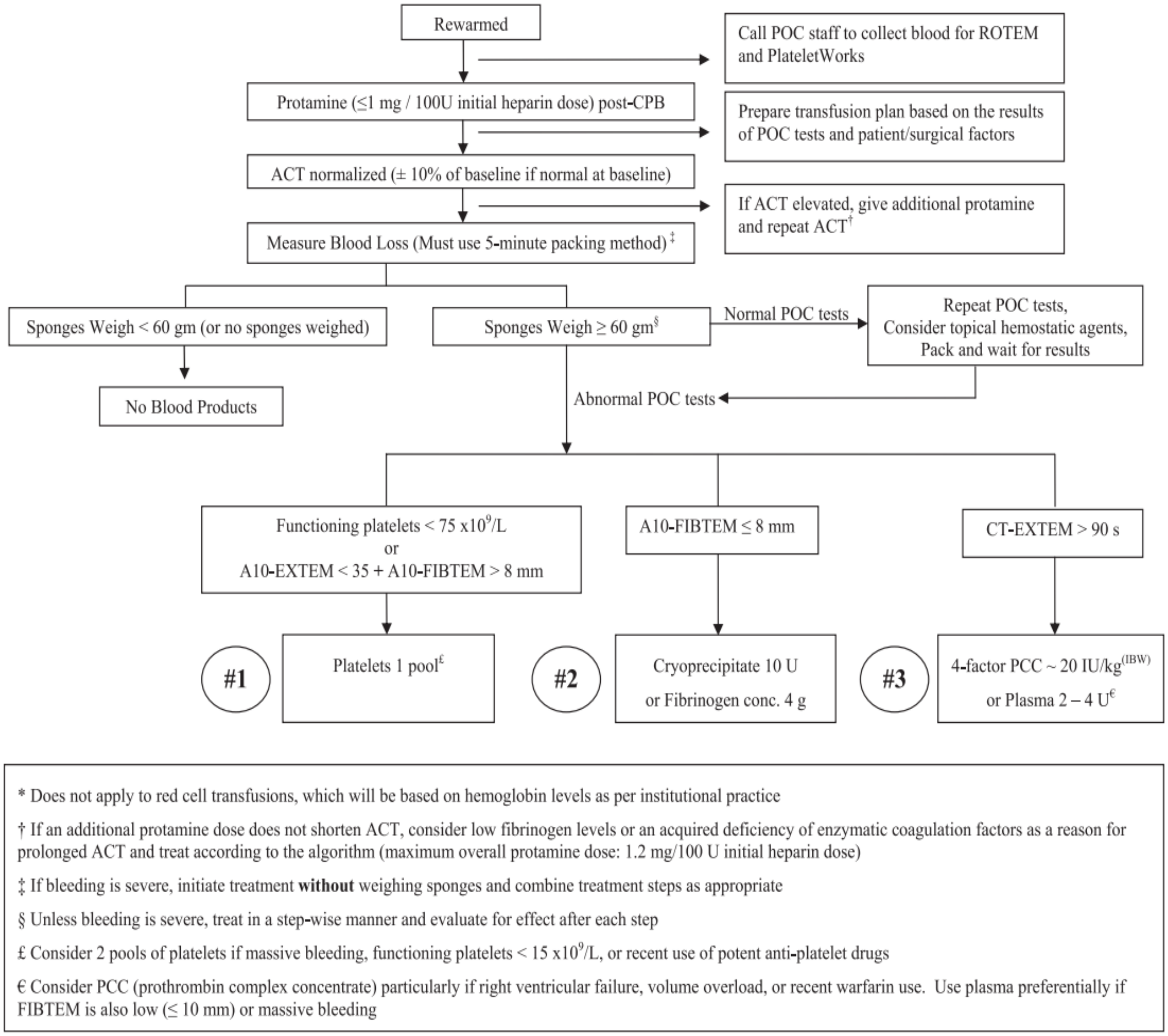

Figure 2. Cardiac Surgery Blood Transfusion Algorithm from TACS. Point-of-care (POC) tests consisted of ROTEM (Thromboelastometry; Tem International GmbH, Munich, Germany) and Plateletworks (Helena Laboratories, Beaumont, TX) systems. ROTEM measures included A10-EXTEM (clot amplitude at 10 minutes with the ROTEM extrinsic pathway assay: 90 s implies poor clot initiation possibly because of reduced coagulation factor levels or reduced thrombin generation). Functioning platelet count was obtained by the Platelet Works assay. ACT indicates activated clotting time; CPB, cardiopulmonary bypass; IBW, ideal body weight.

6.1 Study Procedure

6.1.1 Prior to Enrolment

First coagulation factor replacement order from the surgical team received at the blood bank

The blood bank technologist will confirm the following:

- Eligibility criteria are met
- Coagulation measures (coagulation profile) are available or have been collected or will be collected before the IMP is infused

The blood bank technologist will then randomize patients to Octaplex or FP according to the randomization schedule, and prepare and release the product

Subsequent coagulation factor replacement orders for 24 hours from randomization

- The MLT will ensure Coagulation measures (ROTEM or INR) are available, have been collected, or will be collected before IMP administration.
- If no, ask for sample to be sent to the laboratory prior to and after (within 75 min of) the administration of IMP

Time of IMP infusion in the Operating Room (OR)

The following information will be collected:

- Start time of IMP administration.
- Time of coagulation analyses (coagulation profile).

6.1.2 Visit 1: First post-randomization visit (0 to 24 hours after randomization)

For any specified activity that cannot be completed on this visit, additional visits will be made until all study data are obtained.

- Obtain consent from patient or surrogate
- Collect baseline data
    - Demographics
    - Medical history
    - Preoperative concomitant medications
- Collect surgical data
    - Intraoperative concomitant medications
    - CPB start and end times
    - Cross-clamp time
    - Circulatory arrest start and end times
    - Fluid in- and output monitoring
    - Inotropes and vasopressors
- Collect laboratory assessments where available as part of routine care
    - Chemistry (sodium, potassium, chloride, bicarbonate, pH)

  - Hematology (complete blood count)
  - Coagulation profile before and after IMP administration
  - Safety labs (creatinine, liver function tests [ALT, ALP], bilirubin, troponin)
- Collect all transfusion and hemostatic agents (number and timing) for the 24 hour period after randomization
- Collect bleeding components using the e-CABG and UDPB criteria (1, 72) as well as a modified version of the UDPB not including PCC and FP requirements
- Collect extubation time
- Collect concomitant medications
- Collect AEs and SAEs

6.1.3 Visit 2: Postoperative days 2-7 (or at discharge if earlier)

- Collect daily laboratory assessments where available as part of routine care
  - Chemistry (sodium, potassium, chloride, bicarbonate, pH)
  - Hematology (complete blood count)
  - Coagulation profile
  - Safety labs (creatinine, liver function tests [AST, ALT], bilirubin, troponin)
- Collect transfusion requirements
- Collect extubation time (if applicable)
- Collect length of stay in the ICU and hospital (if applicable)
- Collect AEs and SAEs

6.1.4 Visit 3: Postoperative day 28 (in person if in hospital or by phone)

- Collect AEs and SAEs
- Collect extubation time (if applicable)
- Collect length of stay in the ICU (if applicable)
- Collect length of stay in the hospital (if hospital stay is extended)
- Collect concomitant medications
- Document patient outcome at 28-days (still in hospital; dead or alive)

After Visit 3 or on POD-28, the clinical study is considered completed for the patient. No further study-related assessments will be performed, unless safety concerns (e.g., ongoing AEs) require follow-up.

6.1.5 Time Windows Used in this Study, including Tolerances

In this study, the following time windows and tolerances apply:

Table 3 Time Windows Used in this Study

Time point	Time stated	Tolerance
**Blood sampling**	Before IMP administration	Up to 75 minutes
	After IMP administration	Up to 75 minutes

6.2 Duration of Study

6.2.1 Planned Duration for an Individual Patient

The **duration of the treatment period** is 24 hours from time of randomization.

The **duration of the study** for an individual patient is 28 days from patient enrolment when patients will be contacted in person or by phone post-discharge.

6.2.2 Planned Duration for the Study as a Whole

The study will be considered completed when 120 patients are randomized and have finalized day 28. It is estimated that the study will take approximately 9 months for recruitment.

6.2.3 Premature Termination of the Study

The Sponsor, in consultation with the IDSMC, reserves the right to terminate the study at any time. In this event, any necessary procedures will be arranged on an individual study basis after review and consultation by both parties. In terminating the study, the Investigators will ensure that adequate consideration is given to the protection of the patients' interests.

Regulatory authorities and research ethics boards (REBs) will be informed in accordance with national regulations.

Early termination of the study as a whole or by centre may apply for the following reasons:

Early Termination of the Entire Clinical Study

At any time, the study will be terminated prematurely if:

New toxicological or pharmacological findings or safety reports invalidate the earlier positive benefit-risk-assessment.

Early Termination at an Individual Study Centre

At any time, the study can be terminated at an individual centre if:

- The centre cannot comply with the requirements of the protocol.
- The centre cannot comply with GCP standards.
- The a priori determined required recruitment rate is not met.

7 ASSESSMENTS AND METHODS

7.1 Baseline Data

The baseline information and medical history will be recorded during Visit 1, i.e., as soon as possible after randomization.

7.1.1 Demographic and Baseline Characteristics

The demographic and baseline characteristics are sex, age, height, weight, and Body Mass Index (BMI).

7.1.2 Medical History and Prior/Concomitant Medications

The medical history will be obtained by interviewing the patient or from the medical records.

Prior and concomitant medications will be obtained.

7.2 Study Assessments

7.2.1 Surgical and Surgery-Related Data

The following surgical data will be collected: details of procedure, CPB duration, CPB start and end times, cross-clamp duration, circulatory arrest duration, fluid intake and output, any medications administered, hemodynamic support (e.g., IABP), as well as any blood conservation methods used (e.g., hemoconcentration, retrograde prime, cell salvage).

In addition, extubation time, ICU length of stay, and hospital length of stay will be documented.

7.2.2 Transfusion Data

Details of all blood products and hemostatic agents released from the blood bank and transfused will be collected from the blood bank databases. These include allogeneic blood products (RBCs, pooled or apheresis platelets, and plasma) and albumin. Other hemostatic agents include: desmopressin, fibrinogen concentrates, rFVIIa, idarucizumab, and andexanet alpha.

7.2.3 Bleeding Data

The comparison of ‘major’ bleeding based on the validated e-CABG and universal definition of perioperative bleeding (UDPB) in cardiac surgery (1, 72) (Table 4) will be assessed. The UDPB is a multistage definition for perioperative bleeding based on easily measured clinical end points, including total blood loss from chest tubes within 12 hours, allogeneic blood products transfused, surgical re-exploration including cardiac tamponade, delayed sternal closure, and the need for salvage treatment.

Depending on these components, bleeding is graded as insignificant, mild, moderate, severe, or massive. (Table 4) (72).

Table 4 Bleeding categories according to the UDPB in adult cardiac surgery (if different categories indicate mixed definitions of bleeding, the worst definition applies) (72)

Bleeding definition	Postoperative chest tube blood loss within 12 h (mL)	RBC (units)	FP (units)	PLT (units)	Cryoprecipitate	PCCs	rFVIIa	Reexploration /tamponade
Class 0 (insignificant)	<600	0	0	0	No	No	No	No
Class 1 (mild)	601–800	1	0	0	No	No	No	No
Class 2 (moderate)	801–1000	2–4	2–4	Yes	Yes	Yes	No	No
Class 3 (severe)	1001–2000	5–10	5–10	N/A	N/A	N/A	No	Yes
Class 4 (massive)	>2000	>10	>10	N/A	N/A	N/A	Yes	N/A

FP, frozen plasma; N/A, not applicable; PCC, prothrombin complex concentrate; PLT, platelets; rFVIIa, recombinant activated factor VII; UDPB, universal definition of perioperative bleeding.

To remove any potential bias from PCC and/or FP dosing, as their use is dictated by the randomization schedule, a modified version of the UDPB will also be applied, with the following components of the score omitted: transfusion of PCC, and transfusion of FP.

7.3 Laboratory Assessments

7.3.1 Test Parameters and Laboratories (Only if performed as part of standard of care)

Table 5 summarizes all test parameters and the laboratories responsible for analysis.

Table 5 Test parameters and laboratories

Test	Material needed	Responsible laboratory
**Coagulation profile**		
(PT, PTT, INR, fibrinogen activity via Clauss assay)	Citrated blood	Local
ROTEM EXTEM CT	Citrated blood	Local
ROTEM EXTEM MCF	Citrated blood	Local
**PlateletWorks**	Fresh blood	Local
**Hematology – standard panel as per local lab**	Citrated blood	Local
**Clinical chemistry – standard panel as per local lab**	Serum	Local
**Safety labs**		
Troponin	Serum	Local
ALT/ALP	Serum	Local
Bilirubin	Serum	Local
Creatinine	Serum	Local

ALT, alanine aminotransferase; ALP, alkaline phosphatase; CT, clotting time; MCF, maximum clot firmness; PT, prothrombin time; PTT; partial thromboplastin time; ROTEM, thromboelastometry

7.3.2 Blood Sampling

All blood sampling will be performed as per standard practice at the local institution.

The *actual* time of blood sampling will be recorded in the CRF. If the draw time is not recorded for samples processed in the laboratory, the draw time will be estimated based on the received time in the laboratory minus 10 minutes. For ROTEM and PlateletWorks, if the exact draw time is not recorded, the time for start of testing will be used for draw time.

7.3.3 Citrated and Fresh Blood

Citrated and fresh blood as required by the local laboratory will be collected and processed in accordance with local requirements.

7.3.4 Serum

Serum will be obtained for the determination of clinical chemistry and safety labs (ALT, ALP, creatinine, bilirubin, troponin), where a serum blood sample has been collected.

7.3.5 Recording of Clinically Significant Abnormal Laboratory Values as AEs/ADRs

Other than abnormal laboratory values due to the underlying condition, the Investigator must assess the clinical significance of abnormal laboratory values outside the specified normal range (see **Section 7.4**). Any clinically significant abnormalities will be documented. All specified clinically significant abnormalities will be documented as AEs/SAEs and investigated.

Additional tests and other evaluations required to establish the significance or etiology of specified abnormalities or to monitor the course of an AE will be obtained if clinically indicated. Follow-up will persist until resolution or up to the Study Completion Visit, whichever occurs first.

7.4 Safety Assessments

7.4.1 Assessments for Safety Endpoints

The following drug safety information will be collected:

- AEs and SAEs temporally associated with the administration of IMP (for definitions and reporting requirements, see **Sections 7.4.2, 7.4.3,** and **7.4.4**).
- Pregnancies, drug overdose, interaction, medication error, and lack of efficacy (see **Section 7.4.5**).

7.4.2 Adverse Events (AEs)

Definitions

Adverse event (AE): An AE is any untoward medical occurrence in a study patient receiving an IMP and which does not necessarily have a causal relationship with this treatment. An AE can therefore be any unfavorable and unintended sign (including an abnormal laboratory finding), symptom, or disease temporally associated with the use of an IMP, whether or not related to the IMP.

Adverse drug reaction (ADR): An ADR is any noxious and unintended response to an IMP related to any dose. The phrase 'response to an IMP' means that a causal relationship between the IMP and an AE carries at least a reasonable possibility, i.e., the relationship cannot be ruled out.

Other significant AEs: Any marked laboratory abnormalities or any AEs that lead to an intervention, including withdrawal of drug treatment, dose reduction, or significant additional concomitant therapy.

Withdrawal due to AE/ADR: AE/ADR leading to discontinuation of treatment with IMP. Any such events will be followed up by the Investigator until the event is resolved or until the medical condition of the patient is stable. All follow-up information collected will be made available to the Principal Investigator (Sponsor).

Collection of AEs

The condition of the patient will be monitored throughout the study. At each visit, whether scheduled or unscheduled, AEs will be elicited using a standard non-leading question such as "How have you been since the last visit/during the previous study period?" In addition, the research assistant will check the patient records for any documented event.

Any AE or ADR which occurs during the study will be noted in detail on the appropriate pages of the CRF. If the patient reports several signs or symptoms representing a single syndrome or diagnosis, the diagnosis should be recorded in the CRF. The Investigator will grade the severity of all AEs or ADRs (mild, moderate, or severe), the seriousness (non-serious or serious), and the likelihood that they were related to the IMP (causality). The Sponsor will be responsible for assessing the expectedness of each ADR (expected or unexpected).

Diseases, signs and symptoms, and/or laboratory abnormalities already present before the first administration of IMP will not be considered AEs unless an exacerbation in intensity or frequency (worsening) occurs.

The Investigator will provide detailed information about any abnormalities and about the nature of and reasons for any action taken as well as any other observations or comments that may be useful for the interpretation and understanding of an AE or ADR.

Severity of AEs

The intensity/severity of AEs will be graded as follows:

Mild: an AE, usually transient, which causes discomfort but does not interfere with the patient's routine activities

Moderate: an AE which is sufficiently discomforting to interfere with the patient's routine activities

Severe: an AE which is incapacitating and prevents the pursuit of the patient's routine activities

The grading of an AE is up to the medical judgment of the Investigator and will be decided on a case-by-case basis.

Causality of AEs

All AEs will be assessed by a blinded Investigator as to whether they can be explained by the patient's underlying condition or surgical course. If they are not explainable, then the Investigator will make a determination of the relationship of the AE with the IMP as follows:

Probable: reports including good reasons and sufficient documentation to assume a causal relationship, in the sense of plausible, conceivable, likely, but not necessarily highly probable. A reaction that follows a reasonable temporal sequence from administration of the IMP; or that follows a known or expected response pattern to the suspected medicine; or that is confirmed by stopping or reducing the dosage of the medicine and that could not reasonably be explained by known characteristics of the patient's clinical state.

Possible: reports containing sufficient information to accept the possibility of a causal relationship, in the sense of not impossible and not unlikely, although the connection is uncertain or doubtful, for example because of missing data or insufficient evidence. A reaction that follows a reasonable temporal sequence from administration of the IMP; that follows a known or expected response pattern to the suspected medicine; but that could readily have been produced by a number of other factors.

Unlikely: reports not following a reasonable temporal sequence from IMP administration. An event which may have been produced by the patient's clinical state or by environmental factors or other therapies administered.

Not related (unrelated): events for which sufficient information exists to conclude that the etiology is unrelated to the IMP.

Unclassified: reports which for one reason or another are not yet assessable, e.g., because of outstanding information (can only be a temporary assessment).

Classification of ADRs by Expectedness

ADRs will be classified by the Sponsor as either expected or unexpected:

Expected: an ADR that is listed in the current edition of the Product Monograph.

Unexpected: an ADR that is not listed in the current edition of the Product Monograph, or that differs because of greater severity or greater specificity.

Outcome of AEs

The outcome of all reported AEs has to be documented as follows:

1. Recovered, resolved
2. Recovering, resolving
3. Not recovered, not resolved (by Study Completion visit)
4. Recovered, resolved with sequelae
5. Fatal
6. Unknown

NOTE: A patient's **death** per se is not an event, but an outcome. The event which resulted in the patient's death will be fully documented and reported.

Action(s) taken

AEs requiring action or therapy must be treated with recognized standards of medical care to protect the health and well-being of the patient. Appropriate resuscitation equipment and medicines must be available to ensure the best possible treatment in an emergency situation.

The action taken by the Investigator must be documented:

a) ***General actions taken in the event of an AE***
- None
- Medication (other than IMP) or other (e.g., physical) therapy started
- Test performed
- Other (to be specified)

b) ***IMP-related actions taken in the event of an AE***
- None
- Product withdrawn
- Dose reduced

The Investigator will follow up on each AE until it has resolved or until the medical condition of the patient has stabilized. Any relevant follow-up information will be reported to the Principal Investigator (Sponsor).

7.4.3 Serious Adverse Events (SAEs)

A **serious AE (SAE)** is any untoward medical occurrence that at any dose:

- results in death,
- is life-threatening (see below),
- requires hospitalization or prolongation of existing hospitalization,
- results in persistent or significant disability/incapacity,
- is another important medical event.

In this study a number of SAEs are expected because of the nature of the surgery. These events will not be considered as SAEs in this study.

NOTE: The term 'life-threatening' refers to an event in which the patient was, in the view of the reporting Investigator, at immediate risk of death at the time of the event; it does not refer to an event which may hypothetically have caused death had it been more severe.

In deciding whether an AE/ADR is serious, medical judgment will be exercised. Thus, important AEs/ADRs that are not immediately life-threatening or do not result in death or hospitalization but may jeopardize the patient or may require intervention to prevent one of the other outcomes listed in the definitions above should also be considered serious.

In addition, although not classified under the seriousness criteria, all suspected transmissions of an infectious agent will be reported as an SAE. A suspected virus transmission means that virus antigen has been detected in the patient. A passive transmission of antibodies alone does not constitute a suspected virus transmission.

7.4.4 SAE Reporting Timelines

All SAEs, whether or not they are suspected to be related to study treatment (Appendix 15.1), will be reported within 24 hours of recognition to the Sponsor by telephone, fax, or email:

Keyvan Karkouti MD
Department of Anesthesia
Toronto General Hospital
200 Elizabeth Street, 3EN
Toronto, ON
M5G 2C4
Canada

Phone: 1-416-340-5164
Fax: 1-416-340-3698
Email: keyvan.karkouti@uhn.ca

In addition, all serious adverse events related to *Octaplex* will be reported within 1-month of recognition of the event to:

Octapharma's Corporate Drug Safety Unit
OCTAPHARMA Pharmazeutika Produktionsges.m.b.H.
Oberlaaer Strasse 235, 1100 Vienna, Austria
Fax: +43 1 61032-9949
Email: cdsu@octapharma.com

24 hours emergency telephone number: +43 1 40 80 500

Waivers from the SAE Reporting Requirement

Waivers from the SAE reporting requirement include surgeries that are elective or were planned before study entry or prolongations of existing hospitalizations for economic or social, but not medical, reasons. Such surgeries or prolongations of hospitalizations should not be considered SAEs.

7.4.5 Other Relevant Safety Information

Pregnancies

Patients who are known to be pregnant will not be included in the study. In patients of reproductive age, pregnancy is ruled out prior to the cardiac surgery as part of standard of care.

Overdose, interaction and medication error

The following safety relevant information should be reported as an AE or, if the reaction fulfills one of the criteria for seriousness, as an SAE.

a) Drug overdose

An overdose is a deliberate or inadvertent administration of a treatment at a dose higher than specified in the protocol and higher than the known therapeutic dose that is of clinical relevance. The reaction must be clearly identified as an overdose.

b) Drug interaction

A drug interaction is a situation in which a substance or medicinal product affects the activity of an IMP, i.e., increases or decreases its effects, or produces an effect that none of the products would exhibit on its own. The reaction must be clearly identified as a drug interaction.

c) Medication error

A medication error involves the inadvertent administration or unintended use of a medicinal product which may be caused by the naming, presentation of pharmaceutical form/packaging, or instructions for use/labeling. The reaction must be clearly identified as a medication error.

8 DATA HANDLING AND RECORD KEEPING

8.1 Documentation of Data

8.1.1 Source Data and Records

Source data are defined as all information related to clinical findings, observations, or other activities in the study, written down in original records or certified copies of original records, allowing reconstruction and evaluation of the clinical study.

The Investigator will maintain adequate source records (e.g., case histories or patient files for each patient enrolled). Source records should be preserved for 10 years, as required by local regulations.

For each patient enrolled, the Investigator will indicate in the source record(s) that the patient participates in this study.

For data capture, a validated Electronic Data Capture (EDC) system providing an electronic CRF (eCRF) application will be used.

All data entered in the eCRF must be supported by source data in the patient records.

The Investigator will permit study-related monitoring, audit(s), REB review(s), and regulatory inspection(s), by providing direct access to the source data/records.

The Investigator may authorize site staff (e.g., sub-investigators, clinical research coordinators/assistants, nurses) to enter study data into the eCRF. This must be documented in the Delegation of Authority Log signed by the Investigator.

8.1.2 Case Report Forms

Authorized study site staff (e.g., blood bank technologist, research coordinator/assistant) will be responsible for completing an eCRF record for each patient enrolled. All site personnel will be trained on completion of a patient's eCRF. The site is also provided with the approved eCRF Completion Guidelines which will assist in data entry and data issues/questions. Additional site training may be provided as refreshers throughout the study, if needed. All persons allowed to enter or to change eCRF data must be listed in the Delegation of Authority Log.

For each patient enrolled, an eCRF record will be completed within the EDC system and finally approved by the Investigator or an authorized sub-investigator.

Prior to operational use, the eCRF application will be validated. All site personnel will be trained on the EDC system and study specific eCRFs prior to receiving access to the live system for data entry.

8.1.3 Data Validation Procedures

Monitors will perform source data verification (SDV) as defined for the study and described in the study Monitoring Plan.

If any errors or discrepancies in the eCRFs are found during data entry or review, discrepancies will be generated programmatically within the EDC system, and ‘manual’ queries will be generated by either a monitor or Data Management.

Discrepancies and queries can only be corrected by the Investigator(s) or other authorized site personnel. An audit trail documents all changes to the data over the entire study period. If the reason for a change is not obvious, a comment must be supplied in the query’s response, stating the reason for the change, prior to closing. The study monitor should provide guidance to Investigator(s) and the Investigator(s)’ designated representatives on making such corrections.

Once queries have been resolved by the site staff, the resolutions are assessed by Data Management. If the query response provided confirms the data as correct, the discrepancy will be closed. If the response does not adequately address the question raised, a new query will be issued for further clarification.

Manual checks are performed and programs are run throughout the study until the data is clean and the database is ready for lock. All discrepancies will be resolved prior to database lock. There will be a final run of the programmed checks to ensure all discrepancies are closed out, SDV will be confirmed as complete by the monitor, and all eCRFs will be approved by the Investigator prior to database lock.

8.2 Information to Investigators

A Product Monograph will be handed out to the Investigator before the start of the study. The Product Monograph contains all information in the Sponsor’s possession necessary for the Investigator to be fully and accurately informed about the safety of *Octaplex*.

The Product Monograph will be updated at regular intervals by Octapharma and whenever relevant new information concerning the IMP becomes available. This will be delivered by Octapharma to the Principal Investigator/Sponsor who will distribute to the approved study sites.

The Investigator will be informed about the methods for rating relevant study outcomes and for completing CRFs to reduce discrepancies between participating Investigator and study sites.

The Investigator will be kept informed of important data that relate to the safe use of the IMP as the study proceeds.

8.3 Responsibilities

At each study site the Investigator is accountable for the conduct of the clinical study. Responsibilities may be delegated to appropriately qualified persons.

A Delegation of Authority Log will be filled in and signed by the Investigator. In accordance with this authority log, study site staff (e.g., sub-investigators, nurses) are authorized to perform tasks relating to the study.

8.4 Investigator’s Site File

At each study site, the Investigator is responsible for maintaining all records to enable the conduct of the study to be fully documented. Essential documents as required by GCP guidelines and regulations (e.g., copies of

the protocol, study approval letters, all original informed consent forms, site copies of all CRFs, drug dispensing and accountability logs, correspondence pertaining to the study, etc.) should be filed accurately and kept by the Investigator for the maximum period of time required by local regulations.

The Investigator is responsible for maintaining a confidential patient identification code list, which provides the unique link between named source records and CRF data for the Sponsor. The Investigator must arrange for the retention of this confidential list for the maximum period of time required by local regulations.

No study document should be destroyed without prior written agreement between the Investigator and the Sponsor. Should the Investigator elect to assign the study documents to another party, or move them to another location, the Sponsor must be notified in writing.

8.5 Provision of Additional Information

On request, the site investigators will supply the Sponsor or designate, such as the monitors with additional data relating to the study, or copies of relevant source records, ensuring that the patient's confidentiality is maintained. This is particularly important when CRFs are illegible or when errors in data transcription are encountered. In case of particular issues or governmental queries, it is also necessary to have access to the complete study records, provided that the patient's confidentiality is protected in accordance with applicable regulations.

8.6 Independent Data Safety Monitoring Committee

An IDSMC will be established by the Sponsor. The IDSMC will be composed of recognized experts in the field of statistics, perioperative medicine, and hematology who are not actively recruiting patients.

A written study-specific charter will define in detail the composition, responsibilities, and procedures of the IDSMC.

9 STATISTICAL METHODS AND SAMPLE SIZE

The statistical analysis will be delegated under an agreement of transfer of responsibilities to an external statistician. Details on planned descriptive and exploratory analyses will be provided in a separate Statistical Analysis Plan (SAP).

9.1 Determination of Sample Size

This pilot study will include 120 randomized patients, which, accounting for randomized but untreated patients and patients who withdraw in both arms, is expected to provide at least 100 evaluable patients, with 50 in each arm. This population is expected to be clinically sufficient to allow selection of an appropriate endpoint for the confirmative Phase 3 study and to determine feasibility. No statistical sample size estimation has been involved.

9.2 Statistical Analysis

For the statistical analysis of the efficacy parameters the following analysis populations will be considered:

The statistical analyses will be performed on the intention to treat (ITT) population, which will include all randomized and treated patients who consent to the study. Randomized patients who did not undergo cardiac surgery or received no IMP will be excluded from the analysis. It is anticipated that <20% of patients will fall into these categories. In the event that a patient receives treatment that is not in concordance with the randomization schedule, the treatment group will be defined according to the randomization (rather than the actual treatment received).

The per-protocol (PP) population: This analysis population will consist of all patients in the ITT population, excluding patients with important protocol deviations that may affect the statistical analysis. The following patients will be excluded by default:

- Patients who receive an IMP different to the IMP assigned by randomization
- Patients who receive less than 80% of the planned dose
- Patients who significantly violate inclusion/exclusion criteria

A final decision about the classification of protocol deviations and their consequences regarding assignment of patients to analysis populations will be made during the data review meeting prior to the final analyses. Decisions and outcome will be approved by the Principal Investigator (Sponsor) in consultation with the funder (Octapharma).

The ITT analysis population is considered the primary population for analysis of the efficacy objectives.

Descriptive statistics with 95% confidence intervals will be used to describe the data. Simple tests of comparison will be used to compare outcomes between the two groups. The summary tables and exploratory inferences will be chosen according to the scaling level of the measurements, e.g. frequency tables and Chi-square tests for categorical responses, sampling statistics and F-tests for continuous data.

9.2.1 Safety Analysis Plan

The safety analysis population (SAF) will include all patients who received at least one dose of the IMP who consent to the study (if no randomization errors occur, this will be the same population as the ITT population). Safety outcomes will be analyzed by presenting point estimates and two-sided 95% CIs in addition to descriptive statistics.

To ensure that the safety reporting is complete, all hemostatic therapy and Grades 3 and 4 SAE (as per the FDA criteria: severe or life-threatening) data will be collected in cases where consent for remaining in the study cannot be obtained.

Adverse events, including thromboembolic and other events of special interest

AEs will be coded according to the latest Medical Dictionary for Regulatory Activities (MedDRA) version as specified in the Data Management Plan. The analysis will focus on treatment emergent adverse events (TEAEs), i.e., AEs that started or worsened after start of infusion with IMP.

All TEAEs, related TEAEs (i.e., AEs unlikely, probably or possibly related to the IMP), and serious TEAEs will be summarized and tabulated according to primary system organ class and preferred term. TEAEs leading to death and TEAEs resulting in withdrawal from the study will be tabulated using frequency tables if a reasonable number of events of this type are observed.

Analogous frequency tables for thromboembolic events (TEEs, identified using MedDRA SMQs), composite events, TRALI and TACO will be provided separately.

Patient listings will be provided for patients with SAEs, TEEs, AEs leading to withdrawal from the study, and AEs leading to death. Listings for non-treatment emergent adverse events, e.g. for patients enrolled but not randomized will be provided separately.

Mortality

The number of patients who died will be summarized. A possible difference between treatment groups will be estimated by a risk ratio with 95% CI. Kaplan-Meier estimates for the time to death distribution will be calculated and graphically presented if a sufficient number of cases have been registered.

Routine laboratory data

All laboratory values will be classified as normal or abnormal according to the laboratories' normal ranges and indicated as clinically significant or not clinically significant by the Investigator on specified ranges. The following approaches will be taken for each laboratory parameter for the statistical analysis:

- Quantitative data will be examined for trends using descriptive analysis (number of patients, number of missing values, mean, SD, median, quartiles, minimum, maximum) of actual values at each scheduled time point and changes from baseline to each scheduled time point. Mean concentration vs time profiles will be plotted for quantitative data
- Qualitative data based on reference ranges will be described according to the categories (i.e., low, normal, high)

- Shift tables illustrating changes with respect to the laboratories' normal ranges between baseline and a defined scheduled time point. Scatterplots between scheduled time points of lab values with normal ranges will be used for visual illustration.
- Number and frequency of patients with clinically significant laboratory values. A separate patient listing will be provided

9.2.2 Subgroup Analysis

Due to the exploratory character of this pilot study efficacy and selected safety results will further be investigated within subgroups.

This will be done to detect possible relationships that procedural or patient characteristics may have on the response parameters.

If the levels of subgroups (e.g. surgery types) are too numerous to form subgroups of sufficient size, the influence of the characteristic will be studied using model-based exploratory analyses (e.g. logistic regression, Analysis of Covariance, Proportional hazards regression).

Details on the subgroup and model-based inferences will be given in a Statistical Analysis Plan that will be finalized before the Data Review Meeting.

9.2.3 Handling of Missing Data

In general, missing data will not be imputed. Due to the nature of the study, important variables are expected to have few missing data.

9.3 Randomization, Stratification, and Code Release

Eligible patients will be randomly assigned to receive either *Octaplex* or FP. Randomization lists using a permuted-block, randomization scheme (stratified by site) will be prepared by the biostatistician. Sealed randomization envelopes based on the randomization lists will then be provided to the blood banks of the participating centres who will be responsible for providing the IMP.

Patients will be identified using a sequential numbering system within the centre. Randomization will then be performed in sequential order of the patient IDs.

10 ETHICAL/REGULATORY, LEGAL AND ADMINISTRATIVE ASPECTS

10.1 Ethical/Regulatory Framework

This study will be conducted in accordance with the ethical principles laid down in the Declaration of Helsinki. The study protocol and any subsequent amendment(s) will be submitted to an REB and to the Regulatory Authority. The study will be conducted in compliance with the protocol, GCP guidelines, and applicable regulatory requirements.

The regulatory application or submission for regulatory approval will be made by the Sponsor or designated third party (e.g., CRO).

10.2 Approval of Study Documents

The study protocol, a sample of the debriefing form, any other materials provided to the patients, and further requested information will be submitted by the Sponsor or the Site Investigator to the appropriate REB. The study must be approved by the REB before the patient is exposed to a study-related procedure.

The Sponsor, the Site Investigator and any third party (e.g., CRO) involved in obtaining approval must inform each other in writing that all ethical and legal requirements have been met before the first patient is enrolled in the study.

10.3 Consent Issues

This is a pragmatic trial that compares two coagulation factor replacement sources that are currently within the standard of care for this procedure, is unlikely to pose additional risks to patients, and entails no additional interventions outside of normal clinical care. Moreover, due to the emergency nature of the condition being studied (i.e., bleeding during or after surgery), the trial will include only patients who are incapable of providing informed consent at the time the therapy is needed and in whom delays in obtaining surrogate consent can be severely detrimental to their well-being. In addition, this complication occurs infrequently and cannot be predicted before surgery. Thus, while it is technically possible to obtain informed consent before all surgeries, it is simply ‘impracticable’ to do so for this specific study, thereby rendering such a study simply unfeasible.

Importantly, the study compares two substitutable therapies that are used as part of routine clinical care, and there is no compelling theoretical basis or any types of data that patients would be placed at risk by participating in the study. The results of this study, on the other hand, would have important societal benefits, as it will help the Canadian Blood Services to determine which of the two products should be supplied in the future. If the subsequent confirmative Phase 3 study finds PCC to be non-inferior to FP, it will likely be the treatment of choice because it has a lower theoretical risk of viral transmission, and allows for more accurate and rapid targeted therapy of bleeding.

The study meets the criteria stated in Article 3.7A of the 2014 Tri-Council Policy Statement on the Ethical Conduct for Research Involving Humans for identifying situations in which exceptions may be sought for the requirement to seek prior consent. Thus, we will seek delayed consent from patients (or surrogate decision maker (SDM) where appropriate) as outlined below.

Patient or SDM will be approached for informed consent within 24-72 hours after surgery. They will be provided as much time as necessary to ask any questions and to make their decision. If initially the patient is not capable of providing consent, consent will be obtained only from the SDM. The patient will be re-visited every

few days and consent will be obtained from the patient when appropriate. Consent should be obtained within the 28 day duration for each study patient. Any delays in obtaining consent due to extenuating circumstances need to be clearly documented (e.g., a patient dies soon after surgery in which case initiation of consent process from next-of-kin can be delayed for up to 2-weeks after death). Study data collection will begin only after informed consent is obtained from the SDM or patient.

Study coordinator or assistant will obtain freely given consent (in person or via telephone/-) from each patient (or SDM) after an appropriate explanation of the aims, methods, anticipated benefits, potential hazards, and any other aspect of the study which is relevant to the decision to continue to participate. E-mail will be used as a method of correspondence where requested to send the consent form for review. Consent must be obtained by the patient (or SDM), before the patient is exposed to any further study-related procedures, namely evaluation and data collection. Should the consent be obtained from the SDM and later withdrawn by the patient, no additional data will be collected. To ensure that the safety reporting is complete, all hemostatic therapy and Grades 3 and 4 SAE (as per the FDA criteria: severe or life-threatening) data will be collected in cases where consent for remaining in the study cannot be obtained.

Study coordinator or assistant will explain that the patients/SDM are completely free to withdraw from the study at any time, without any consequences to their future care and without the need to justify. Each patient will be informed that his/her medical (source) records may be reviewed by the study monitor, a quality assurance auditor, or a health authority inspector, in accordance with applicable regulations, and that these persons are bound by confidentiality obligations. In addition to the study protocol, study hospitals will also follow their institutional guidelines and approvals when consenting and collecting data. Their process will be documented in their SOPs. Again, no data will be collected on patients/SDMs who refuse consent. Please refer to Appendix in 15.2 for the consent and data collection guidelines at Toronto General Hospital.

10.4 Protocol Amendments

Any amendments will be submitted to the institutional REB and any authority as required by applicable regulations.

REB approval will, at a minimum, be requested for any change to this protocol which could affect the safety of the patients, the objective or design of the study, any increase in dosage or duration of exposure to the IMP, an increase in the number of patients treated, the addition of a new test or procedure, or the dropping of a test intended to monitor safety.

10.5 Confidentiality of Patient Data

The Investigator will ensure that the patient's confidentiality is preserved. On CRFs or any other documents submitted to the Sponsor, the patients will not be identified by their names, but by a unique patient identifier. Documents not intended for submission to the Sponsor, i.e., the confidential patient identification code list, original consent forms, and source records, will be maintained by the Investigator in strict confidence.

11 QUALITY CONTROL AND QUALITY ASSURANCE

11.1 Periodic Monitoring

The monitor will contact and visit the Investigator periodically to review all study-related source data/records, verify the adherence to the protocol and the completeness, correctness and accuracy of all CRF entries compared to source data. The Investigator will co-operate with the monitor to ensure that any discrepancies identified are resolved.

For this study, the first monitoring visit shall take place shortly after the inclusion of the first patient. Thereafter, monitoring frequency will depend on study progress.

The monitor must be given direct access to source documents (original documents, data and records). Direct access includes permission to examine, analyze, verify, and reproduce any records and reports that are important to the evaluation of the clinical study. Source data will be available for all data in the CRFs, including all laboratory results.

Monitoring will take place as per the monitoring plan.

11.2 Audit and Inspection

The Investigator will make all study-related source data and records available to a qualified quality assurance auditor, REB or regulatory inspectors, after reasonable notice. The main purposes of an audit or inspection are to confirm that the rights and welfare of the patients have been adequately protected, and that all data relevant for the assessment of safety and efficacy of the IMP have been captured.

12 REPORTING AND PUBLICATION

12.1 Clinical Study Report

A clinical study report (in accordance with relevant guidelines) will be prepared by the Sponsor after completion of the study.

12.2 Publication Policy

The results of this study will be published and may be presented at scientific meetings.

In accordance with standard editorial and ethical practice, the Investigator will publish the multicentre data only in their entirety and not as individual centre data. Authorship will be determined by mutual agreement. Any subsequent publications based on subsets of the data will require approval from the Sponsor.

13 LIABILITIES AND INSURANCE

In order to cover any potential damage or injury occurring to a patient in association with the IMP or participation in the study, the Investigators and/or their institutions will contract insurance in accordance with local regulations.

The Investigator is responsible for dispensing the IMP according to this protocol and for its secure storage and safe handling throughout the study.

14 REFERENCES

1. Mariscalco G, Gherli R, Ahmed AB, Zanobini M, Maselli D, Dalen M, et al. Validation of the European Multicenter Study on Coronary Artery Bypass Grafting (E-CABG) Bleeding Severity Definition. Ann Thorac Surg. 2016;101(5):1782-8.

2. Dyke C, Aronson S, Dietrich W, Hofmann A, Karkouti K, Levi M, et al. Universal definition of perioperative bleeding in adult cardiac surgery. J Thorac Cardiovasc Surg. 2014;147(5):1458-63.

3. Leach Bennett J, Blajchman MA, Delage G, Fearon M, Devine D. Proceedings of a consensus conference: Risk-Based Decision Making for Blood Safety. Transfus Med Rev. 2011;25(4):267-92.

4. Karkouti K, Callum J, Wijeysundera DN, Rao V, Crowther M, Grocott HP, et al. Point-of-Care Hemostatic Testing in Cardiac Surgery: A Stepped-Wedge Clustered Randomized Controlled Trial. Circulation. 2016;134(16):1152-62.

5. Stokes ME, Ye X, Shah M, Mercaldi K, Reynolds MW, Rupnow MF, et al. Impact of bleeding-related complications and/or blood product transfusions on hospital costs in inpatient surgical patients. BMC Health Serv Res. 2011;11:135.

6. Karkouti K, Wijeysundera DN, Yau TM, Beattie WS, Abdelnaem E, McCluskey SA, et al. The independent association of massive blood loss with mortality in cardiac surgery. Transfusion. 2004;44(10):1453-62.

7. Karkouti K, Arellano R, Aye T, Dupuis JY, Kent B, Lee TW, et al. Off-label use of recombinant activated factor VII in surgical and non-surgical patients at 16 Canadian hospitals from 2007 to 2010 (Canadian Registry Report). Can J Anaesth. 2014;61(8):727-35.

8. Society of Thoracic Surgeons Blood Conservation Guideline Task F, Ferraris VA, Brown JR, Despotis GJ, Hammon JW, Reece TB, et al. 2011 update to the Society of Thoracic Surgeons and the Society of Cardiovascular Anesthesiologists blood conservation clinical practice guidelines. Ann Thorac Surg. 2011;91(3):944-82.

9. Mehta RH, Sheng S, O'Brien SM, Grover FL, Gammie JS, Ferguson TB, et al. Reoperation for bleeding in patients undergoing coronary artery bypass surgery: incidence, risk factors, time trends, and outcomes. Circ Cardiovasc Qual Outcomes. 2009;2(6):583-90.

10. Karkouti K, Beattie WS, Arellano R, Aye T, Bussieres JS, Callum JL, et al. Comprehensive Canadian review of the off-label use of recombinant activated factor VII in cardiac surgery. Circulation. 2008;118(4):331-8.

11. Karkouti K, Yau TM, Riazi S, Dattilo KM, Wasowicz M, Meineri M, et al. Determinants of complications with recombinant factor VIIa for refractory blood loss in cardiac surgery. Can J Anaesth. 2006;53(8):802-9.

12. Erber WN. Massive blood transfusion in the elective surgical setting. Transfus Apher Sci. 2002;27(1):83-92.

13. Koch CG, Li L, Duncan AI, Mihaljevic T, Loop FD, Starr NJ, et al. Transfusion in coronary artery bypass grafting is associated with reduced long-term survival. Ann Thorac Surg. 2006;81(5):1650-7.

14. Dixon B, Santamaria JD, Reid D, Collins M, Rechnitzer T, Newcomb AE, et al. The association of blood transfusion with mortality after cardiac surgery: cause or confounding? (CME). Transfusion. 2013;53(1):19-27.

15. Ranucci M, Baryshnikova E, Castelvecchio S, Pelissero G, Surgical, Clinical Outcome Research G. Major bleeding, transfusions, and anemia: the deadly triad of cardiac surgery. Ann Thorac Surg. 2013;96(2):478-85.

16. Moulton MJ, Creswell LL, Mackey ME, Cox JL, Rosenbloom M. Reexploration for bleeding is a risk factor for adverse outcomes after cardiac operations. J Thorac Cardiovasc Surg. 1996;111(5):1037-46.

17. Hall TS, Brevetti GR, Skoultchi AJ, Sines JC, Gregory P, Spotnitz AJ. Re-exploration for hemorrhage following open heart surgery differentiation on the causes of bleeding and the impact on patient outcomes. Ann Thorac Cardiovasc Surg. 2001;7(6):352-7.

18. Vivacqua A, Koch CG, Yousuf AM, Nowicki ER, Houghtaling PL, Blackstone EH, et al. Morbidity of bleeding after cardiac surgery: is it blood transfusion, reoperation for bleeding, or both? Ann Thorac Surg. 2011;91(6):1780-90.

19. Surgenor SD, DeFoe GR, Fillinger MP, Likosky DS, Groom RC, Clark C, et al. Intraoperative red blood cell transfusion during coronary artery bypass graft surgery increases the risk of postoperative low-output heart failure. Circulation. 2006;114(1 Suppl):I43-8.

20. Kuduvalli M, Oo AY, Newall N, Grayson AD, Jackson M, Desmond MJ, et al. Effect of peri-operative red blood cell transfusion on 30-day and 1-year mortality following coronary artery bypass surgery. Eur J Cardiothorac Surg. 2005;27(4):592-8.

21. Banbury MK, Brizzio ME, Rajeswaran J, Lytle BW, Blackstone EH. Transfusion increases the risk of postoperative infection after cardiovascular surgery. J Am Coll Surg. 2006;202(1):131-8.

22. Hartmann M, Sucker C, Boehm O, Koch A, Loer S, Zacharowski K. Effects of cardiac surgery on hemostasis. Transfus Med Rev. 2006;20(3):230-41.

23. Parr KG, Patel MA, Dekker R, Levin R, Glynn R, Avorn J, et al. Multivariate predictors of blood product use in cardiac surgery. J Cardiothorac Vasc Anesth. 2003;17(2):176-81.

24. Johansson PI, Solbeck S, Genet G, Stensballe J, Ostrowski SR. Coagulopathy and hemostatic monitoring in cardiac surgery: an update. Scand Cardiovasc J. 2012;46(4):194-202.

25. Despotis G, Eby C, Lublin DM. A review of transfusion risks and optimal management of perioperative bleeding with cardiac surgery. Transfusion. 2008;48(1 Suppl):2S-30S.

26. Bevan DH. Cardiac bypass haemostasis: putting blood through the mill. Br J Haematol. 1999;104(2):208-19.

27. Mann KG, Brummel K, Butenas S. What is all that thrombin for? J Thromb Haemost. 2003;1(7):1504-14.

28. Brummel KE, Paradis SG, Butenas S, Mann KG. Thrombin functions during tissue factor-induced blood coagulation. Blood. 2002;100(1):148-52.

29. Palta S, Saroa R, Palta A. Overview of the coagulation system. Indian J Anaesth. 2014;58(5):515-23.

30. Ghadimi K, Levy JH, Welsby IJ. Perioperative management of the bleeding patient. Br J Anaesth. 2016;117(suppl 3):iii18-30.

31. Chowdary P, Tang A, Watson D, Besser M, Collins P, Creagh MD, et al. Retrospective Review of a Prothrombin Complex Concentrate (Beriplex P/N) for the Management of Perioperative Bleeding Unrelated to Oral Anticoagulation. Clin Appl Thromb Hemost. 2018;24(7):1159-69.

32. Sheffield WP, Bhakta V, Yi QL, Jenkins C. Stability of Thawed Apheresis Fresh-Frozen Plasma Stored for up to 120 Hours at 1 degrees C to 6 degrees C. J Blood Transfus. 2016;2016:6260792.

33. Triulzi D, Gottschall J, Murphy E, Wu Y, Ness P, Kor D, et al. A multicenter study of plasma use in the United States. Transfusion. 2015;55(6):1313-9.

34. Desborough M, Sandu R, Brunskill SJ, Doree C, Trivella M, Montedori A, et al. Fresh frozen plasma for cardiovascular surgery. Cochrane Database Syst Rev. 2015(7):CD007614.

35. Tollofsrud S, Noddeland H, Svennevig JL, Bentsen G, Mollnes TE, Solheim BG. Universal fresh frozen plasma (Uniplas): a safe product in open-heart surgery. Intensive Care Med. 2003;29(10):1736-43.

36. Doussau A, Perez P, Puntous M, Calderon J, Jeanne M, Germain C, et al. Fresh-frozen plasma transfusion did not reduce 30-day mortality in patients undergoing cardiopulmonary bypass cardiac surgery with excessive bleeding: the PLASMACARD multicenter cohort study. Transfusion. 2014;54(4):1114-24.

37. Chai-Adisaksopha C, Hillis C, Siegal DM, Movilla R, Heddle N, Iorio A, et al. Prothrombin complex concentrates versus fresh frozen plasma for warfarin reversal. A systematic review and meta-analysis. Thromb Haemost. 2016;116(5):879-90.

38. Pandey S, Vyas GN. Adverse effects of plasma transfusion. Transfusion. 2012;52 Suppl 1:65S-79S.

39. Holness L, Knippen MA, Simmons L, Lachenbruch PA. Fatalities caused by TRALI. Transfus Med Rev. 2004;18(3):184-8.

40. Narick C, Triulzi DJ, Yazer MH. Transfusion-associated circulatory overload after plasma transfusion. Transfusion. 2012;52(1):160-5.

41. Sarani B, Dunkman WJ, Dean L, Sonnad S, Rohrbach JI, Gracias VH. Transfusion of fresh frozen plasma in critically ill surgical patients is associated with an increased risk of infection. Crit Care Med. 2008;36(4):1114-8.

42. Bjursten H, Dardashti A, Ederoth P, Bronden B, Algotsson L. Increased long-term mortality with plasma transfusion after coronary artery bypass surgery. Intensive Care Med. 2013;39(3):437-44.

43. Khan H, Belsher J, Yilmaz M, Afessa B, Winters JL, Moore SB, et al. Fresh-frozen plasma and platelet transfusions are associated with development of acute lung injury in critically ill medical patients. Chest. 2007;131(5):1308-14.

44. Inaba K, Branco BC, Rhee P, Blackbourne LH, Holcomb JB, Teixeira PG, et al. Impact of plasma transfusion in trauma patients who do not require massive transfusion. J Am Coll Surg. 2010;210(6):957-65.

45. Watson GA, Sperry JL, Rosengart MR, Minei JP, Harbrecht BG, Moore EE, et al. Fresh frozen plasma is independently associated with a higher risk of multiple organ failure and acute respiratory distress syndrome. J Trauma. 2009;67(2):221-7.

46. Percy CL, Hartmann R, Jones RM, Balachandran S, Mehta D, Dockal M, et al. Correcting thrombin generation ex vivo using different haemostatic agents following cardiac surgery requiring the use of cardiopulmonary bypass. Blood Coagul Fibrinolysis. 2015;26(4):357-67.

47. Ghadimi K, Levy JH, Welsby IJ. Prothrombin Complex Concentrates for Bleeding in the Perioperative Setting. Anesth Analg. 2016;122(5):1287-300.

48. Webert KE, Cserti CM, Hannon J, Lin Y, Pavenski K, Pendergrast JM, et al. Proceedings of a Consensus Conference: pathogen inactivation-making decisions about new technologies. Transfus Med Rev. 2008;22(1):1-34.

49. Schubert P, Culibrk B, Karwal S, Slichter SJ, Devine DV. Optimization of platelet concentrate quality: application of proteomic technologies to donor management. J Proteomics. 2012;76 Spec No.:329-36.

50. Raval JS, Waters JH, Seltsam A, Scharberg EA, Richter E, Kameneva MV, et al. Menopausal status affects the susceptibility of stored RBCs to mechanical stress. Vox Sang. 2011;100(4):418-21.

51. Grottke O, Rossaint R, Henskens Y, van Oerle R, Ten Cate H, Spronk HM. Thrombin generation capacity of prothrombin complex concentrate in an in vitro dilutional model. PLoS One. 2013;8(5):e64100.

52. Schochl H, Grottke O, Sutor K, Dony K, Schreiber M, Ranucci M, et al. Theoretical Modeling of Coagulation Management With Therapeutic Plasma or Prothrombin Complex Concentrate. Anesth Analg. 2017;125(5):1471-4.

53. Godier A, Greinacher A, Faraoni D, Levy JH, Samama CM. Use of factor concentrates for the management of perioperative bleeding: guidance from the SSC of the ISTH. J Thromb Haemost. 2018;16(1):170-4.

54. Barco S, Picchi C, Trinchero A, Middeldorp S, Coppens M. Safety of prothrombin complex concentrate in healthy subjects. Br J Haematol. 2017;176(4):664-6.

55. Grottke O, Braunschweig T, Spronk HM, Esch S, Rieg AD, van Oerle R, et al. Increasing concentrations of prothrombin complex concentrate induce disseminated intravascular coagulation in a pig model of coagulopathy with blunt liver injury. Blood. 2011;118(7):1943-51.

56. Mitterlechner T, Innerhofer P, Streif W, Lodl M, Danninger T, Klima G, et al. Prothrombin complex concentrate and recombinant prothrombin alone or in combination with recombinant factor X and FVIIa in dilutional coagulopathy: a porcine model. J Thromb Haemost. 2011;9(4):729-37.

57. Kozek-Langenecker SA, Ahmed AB, Afshari A, Albaladejo P, Aldecoa C, Barauskas G, et al. Management of severe perioperative bleeding: guidelines from the European Society of Anaesthesiology: First update 2016. Eur J Anaesthesiol. 2017;34(6):332-95.

58. American Society of Anesthesiologists Task Force on Perioperative Blood M. Practice guidelines for perioperative blood management: an updated report by the American Society of Anesthesiologists Task Force on Perioperative Blood Management*. Anesthesiology. 2015;122(2):241-75.

59. Fitzgerald J, Lenihan M, Callum J, McCluskey SA, Srinivas C, van Rensburg A, et al. Use of prothrombin complex concentrate for management of coagulopathy after cardiac surgery: a propensity score matched comparison to plasma. Br J Anaesth. 2018;120(5):928-34.

60. Roman M, Biancari F, Ahmed AB, Agarwal S, Hadjinikolaou L, Al-Sarraf A, et al. Prothrombin complex concentrate in cardiac surgery: A systematic review and meta-analysis. Ann Thorac Surg. 2018.

61. Arnekian V, Camous J, Fattal S, Rezaiguia-Delclaux S, Nottin R, Stephan F. Use of prothrombin complex concentrate for excessive bleeding after cardiac surgery. Interact Cardiovasc Thorac Surg. 2012;15(3):382-9.

62. Ortmann E, Besser MW, Sharples LD, Gerrard C, Berman M, Jenkins DP, et al. An exploratory cohort study comparing prothrombin complex concentrate and fresh frozen plasma for the treatment of coagulopathy after complex cardiac surgery. Anesth Analg. 2015;121(1):26-33.

63. Cappabianca G, Mariscalco G, Biancari F, Maselli D, Papesso F, Cottini M, et al. Safety and efficacy of prothrombin complex concentrate as first-line treatment in bleeding after cardiac surgery. Crit Care. 2016;20:5.

64. Biancari F, Ruggieri VG, Perrotti A, Gherli R, Demal T, Franzese I, et al. Comparative analysis of prothrombin complex concentrate and fresh frozen plasma in coronary surgery. Heart Lung Circ. 2018.

65. Fries D. The early use of fibrinogen, prothrombin complex concentrate, and recombinant-activated factor VIIa in massive bleeding. Transfusion. 2013;53 (Suppl 1):91S-5S.

66. Octapharma. Octaplex Product Monograph. 2014.

67. Lubetsky A, Hoffman R, Zimlichman R, Eldor A, Zvi J, Kostenko V, et al. Efficacy and safety of a prothrombin complex concentrate (Octaplex) for rapid reversal of oral anticoagulation. Thromb Res. 2004;113(6):371-8.

68. Majeed A, Agren A, Holmstrom M, Bruzelius M, Chaireti R, Odeberg J, et al. Management of rivaroxaban- or apixaban-associated major bleeding with prothrombin complex concentrates: a cohort study. Blood. 2017;130(15):1706-12.

69. Schulman S, Gross PL, Ritchie B, Nahirniak S, Lin Y, Lieberman L, et al. Prothrombin Complex Concentrate for Major Bleeding on Factor Xa Inhibitors: A Prospective Cohort Study. Thromb Haemost. 2018;118(5):842-51.

70. Kerebel D, Joly LM, Honnart D, Schmidt J, Galanaud D, Negrier C, et al. A French multicenter randomised trial comparing two dose-regimens of prothrombin complex concentrates in urgent anticoagulation reversal. Crit Care. 2013;17(1):R4.

71. Riess HB, Meier-Hellmann A, Motsch J, Elias M, Kursten FW, Dempfle CE. Prothrombin complex concentrate (Octaplex) in patients requiring immediate reversal of oral anticoagulation. Thromb Res. 2007;121(1):9-16.

72. Dyke C, Aronson S, Dietrich W, Hofmann A, Karkouti K, Levi M, et al. Universal definition of perioperative bleeding in adult cardiac surgery. J Thorac Cardiovasc Surg. 2014;147(5):1458-63 e1.

73. Weber CF, Gorlinger K, Meininger D, Herrmann E, Bingold T, Moritz A, et al. Point-of-care testing: a prospective, randomized clinical trial of efficacy in coagulopathic cardiac surgery patients. Anesthesiology. 2012;117(3):531-47.

74. Tao J, Bukanova EN, Akhtar S. Safety of 4-factor prothrombin complex concentrate (4F-PCC) for emergent reversal of factor Xa inhibitors. J Intensive Care. 2018;6:34.

75. Grassetto A, De Nardin M, Ganzerla B, Geremia M, Saggioro D, Serafini E, et al. ROTEM(R)-guided coagulation factor concentrate therapy in trauma: 2-year experience in Venice, Italy. Crit Care. 2012;16(3):428.

76. Ellenberg SS, Temple R. Placebo-controlled trials and active-control trials in the evaluation of new treatments. Part 2: practical issues and specific cases. Ann Intern Med. 2000;133(6):464-70.

77. Rothmann MD, Wiens BL, Chan ISF. Design and analysis of non-inferiority trials. 1st ed. Chow SC, Jones B, Liu JP, Pease KE, Turnbull BW, editors: Chapman & Hall/CRC; 2012 2012.

78. Freedman B. Placebo-controlled trials and the logic of clinical purpose. IRB. 1990;12(6):1-6.

79. Burnouf T, Radosevich M. Nanofiltration of plasma-derived biopharmaceutical products. Haemophilia. 2003;9(1):24-37.

15 APPENDICES

15.1 AE and SAE Flow Chart for Assessment and Reporting

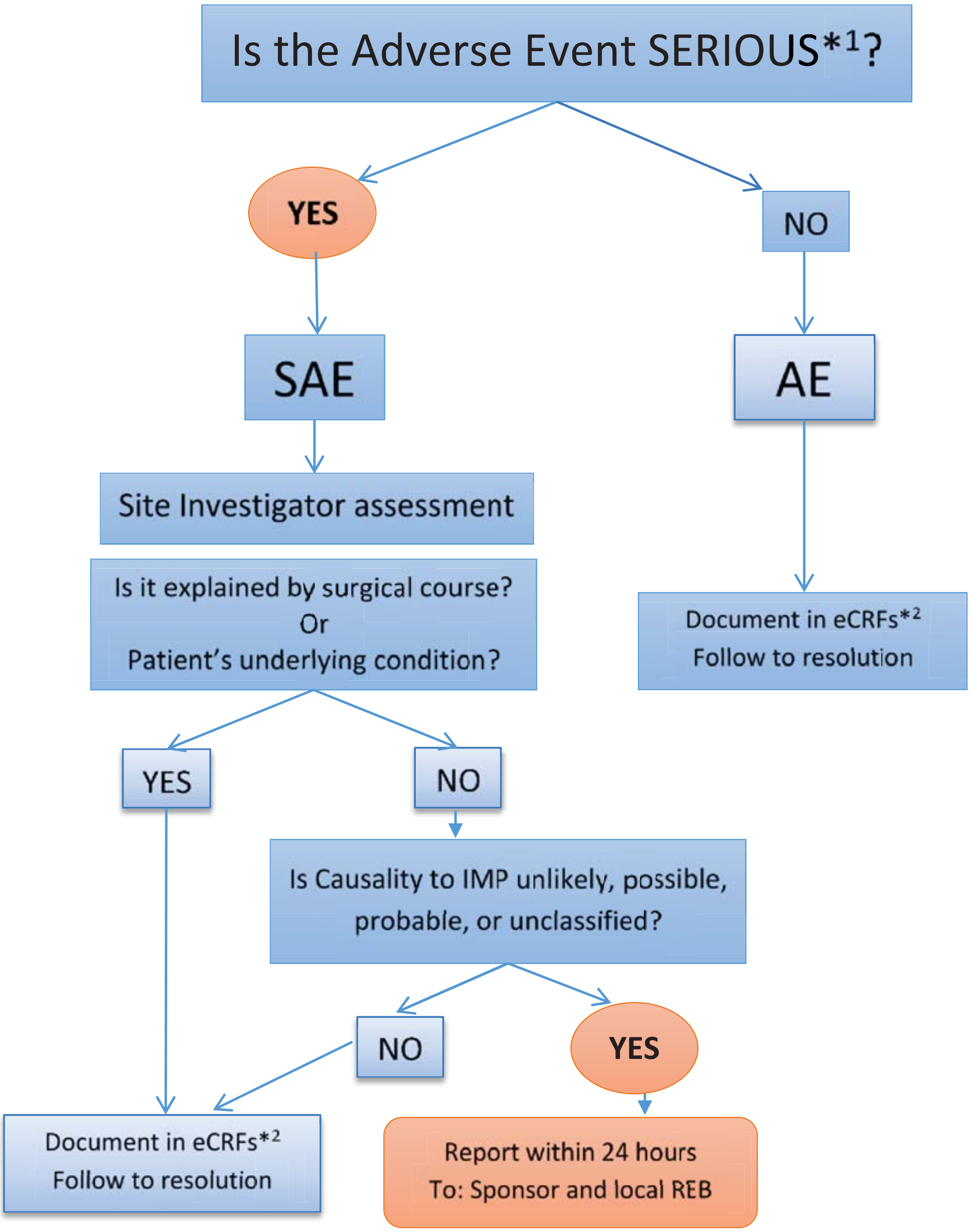

*1 **Serious AE (SAE)** is any untoward medical occurrence that at any dose: results in death, is life-threatening, requires hospitalization or prolongation of existing hospitalization, results in persistent or significant disability/incapacity, is another important medical event.
***2 eCRFs** Case Report Forms **Adverse Events** – Serious Adverse Events Report Form and **Concomitant Medications** Form for each adverse event
Red legend refer to the two conditions of expedited reporting (1) the event is serious and (2) Causality=unlikely, possible, probable, or unclassified to IMP

15.2 Consent and Data Collection Guidelines at TGH

Scenario	What data can be collected
Written consent obtained from patient or Written/telephone consent obtained from SDM	Data can be collected.
Consent obtained initially (from SDM or patient) and later withdrawn (SDM or patient)	Data can be collected up to the date of withdrawal. No data should be collected after withdrawal, not even SAE data.
Consent obtained from SDM and declined from patient once capable of consenting	No data at all should be collected. Patient's decision overwrites the SDM's.
Consent not obtained due to no SDM or patient incapacity to provide consent	All hemostatic therapy and Grades 3 and 4 SAE (as per the FDA criteria: severe or life-threatening) data will be collected.
Consent declined either by patient/ SDM	Nothing can be collected, not even SAE data.

Guidelines to Obtaining Telephone Consent from Patient/SDM

Patient Consent

The following process must be followed when obtaining telephone consent from patients who have been discharged and have requested more time to review the consent form or to discuss the matter with family/friends.

- Contact the patient a week after discharge (unless they have requested to be contacted at a different time point) and ask whether they are agreeable to participate in the study
- If they agree to participate, then data can be collected. If the patient does not agree, nothing can be collected.
- Attempts to make contact with the patient shall be documented and consent shall be obtained in the presence of a witness and documented. If the patient agrees, email communication is permitted.

SDM Consent

The following process must be followed when obtaining telephone consent from the SDM:

- The SDM is first contacted to introduce the study and to establish if they are interested in providing consent on behalf of the patient.
- If they are interested, then they are informed that the team will send the consent form to them and will call back within 2-3 days after the ICF is received. If the SDM agrees, email communication is permitted to send the consent form.
- At the time of the second call, the SDM should be asked whether they agree to participate or not.
- If they agree to participate, then data can be collected. If SDM did not agree, nothing can be collected until the patient is able to give consent.
- Attempts to make contact with the SDM shall be documented and consent shall be obtained in the presence of a witness and documented.

Statistical Analysis Plan (SAP)

Sponsor:	Keyvan Karkouti
Study Title:	FActor REplacement in Surgery<br>Prothrombin complex concentrate versus frozen plasma in bleeding adult cardiac surgical patients: A multicentre, randomized, active-control, pragmatic, Phase 2 pilot study
Protocol Version/Date:	Ver. 1.0; 2019-06-13
SAP Version/Date:	Ver. 2.0; 2020-09-21
Supersedes SAP Version:	Ver. 1.0; 2020-04-30
Appendices (external documents):	1. List of Tables, Listing, Figures (TLFs)

Approval

The Trial Statistician hereby confirms that the SAP was prepared in conformance with the procedures and principles set forth in the indicated protocol version and all established relevant guidelines.

Name<br>Affiliation, Function	Signature:	Date:
Hans-Peter Hucke<br>Ergomed, Trial Statistician		

By signing hereafter, I confirm that this Statistical Analysis Plan adequately describes the statistical analyses to be performed in the context of this study.

Name<br>Affiliation, Function	Signature:	Date:
Keyvan Karkouti<br>Toronto General Hospital<br>Coordinating Investigator		

Revision history

SAP Version	Version date	Reason(s) for change
1.0	2020-04-30	Not applicable. First version.
2.0	2020-09-21	Changes required by discussions during Blinded Data Review Meeting:<br>• Add exploratory analysis to assess the dependence of treatment response on compliance (section 3.6),<br>• Add exploratory analysis to assess endpoints in different time frames<br>• Add another secondary endpoint applying a conservative response definition.<br>• Clarify that ‘other’ medical history will only be listed and only prespecified conditions will be summarized (section 4.4).

TABLE OF CONTENTS

LIST OF ABBREVIATIONS 4
1 Study information 6
1.1 Overall objective 6
1.2 Study design 6
1.3 Planned sample size 7
2 General Information 8
2.1 Background details 8
2.2 Deviations from the trial protocol with regard to statistical analyses 8
2.3 Individual protocol deviations 8
3 Analysis Populations 8
3.1 All Patients Enrolled (APE) Population 9
3.2 Failed Consent Patients (FCP) Population 9
3.3 Safety (SAF) Population 9
3.4 Intention-To-Treat (ITT) Population 9
3.5 Per-Protocol (PP) Population 10
3.6 Subgroup analyses 10
4 Statistical Analyses 10
4.1 Conventions 11
4.1.1 Baseline definition 11
4.1.2 Missing data 11
4.1.3 Pooling of centers 11
4.2 Demographic and other background data 11
4.2.1 Basic description 11
4.3 IMP exposure, compliance 11
4.4 Medical history 11
4.5 Prior and concomitant medication 11
4.6 Efficacy 12
4.7 Safety 14
4.7.1 Adverse events 15
4.7.2 Laboratory variables 16
4.8 Additional safety variables 16
4.9 Interim analyses 17
5 Quality Control 17
6 Derivations and Transformations 18
6.1 Formulas for derived variables 18
6.2 Transformations to be applied 18
7 References 18
Appendices 19
1. List of Tables, Listings, Figures 19

LIST OF ABBREVIATIONS

Abbreviation	Description
ACB	Aortocoronary Bypass
ADR	Adverse Drug Reaction
AE	Adverse Event
ALT	Alanine Aminotransferase
AST	Aspartate Aminotransferase
BMI	Body Mass Index
CI	Confidence Interval
CPB	Cardiopulmonary Bypass
CRF	Case Report Form
CRO	Contract Research Organization
DMP	Data Management Plan
DRM	Data Review Meeting
E-CABG	European Coronary Artery Bypass Grafting
eCRF	Electronic Case Report Form
EDC	Electronic Data Capture
EXTEM	ROTEM Assay Assessing the Extrinsic Coagulation Pathway
FP	Frozen Plasma
GCP	Good Clinical Practice
HIV	Human Immunodeficiency Virus
IABP	Intra-Aortic Balloon Pump
ICU	Intensive Care Unit
IDSMC	Independent Data Safety Monitoring Committee
IMP	Investigational Medicinal Product
INR	International Normalized Ratio
ITT	Intention-To-Treat
IV	Intravenous
MCF	Maximum Clot Firmness
MedDRA	Medical Dictionary for Regulatory Activities
OR	Operating Room
PCC	Prothrombin Complex Concentrate
POD	Postoperative Day
PP	Per-Protocol
PRV	**Pseudorabies Virus**
PT	Prothrombin Time
PTT	Partial Thromboplastin Time
RBC	Red Blood Cell
REB	Research Ethics Board
rFVIIa	Recombinant Activated Factor VII
SAE	Serious Adverse Event
SAF	Safety Analysis Population
SAS	Statistical Analysis Software package
SBV	Schmallenberg Virus
SDM	Surrogate Decision Maker
SDV	Source Data Verification

Abbreviation	Description
TACS	Transfusion Avoidance in Cardiac Surgery
TEAE	Treatment Emergent Adverse Event
TEE	Thromboembolic Event
TLFs	Tables, Listings, Figures
TRALI	Transfusion-Related Acute Lung Injury
TACO	Transfusion-Associated Circulatory Overload
TS	Trial Statistician
UDPB	Universal Definition of Perioperative Bleeding
WFI	Water for Injections
WHO	World Health Organization

1 STUDY INFORMATION

1.1 Overall objective

The main objective of this pilot trial is to inform the design and primary outcome parameter for a definitive Phase 3 trial comparing the efficacy and safety of a Prothrombin Complex Concentrate (PCC; *Octaplex)* versus Frozen Plasma (FP) in bleeding cardiac surgical patients in whom coagulation factor replacement with PCC or FP is ordered according to accepted clinical standards.

1.2 Study design

This is a multicenter, randomized, active-control, pragmatic, Phase 2 pilot study in adult cardiac surgery patients. Two Canadian hospitals (Toronto General and Sunnybrook hospitals, Toronto) will participate and it is estimated that the study will take approximately 9 months to complete.

Patients will be randomized to receive equivalent doses of either PCC (Octaplex) or FP when the blood bank receives the first order for coagulation factor replacement and deems it to be in accordance with accepted clinical standards. Patients will be treated according to their assigned group on the first and second times when coagulation factor replacement is ordered during the treatment period (up to 24 hours after randomization) [see flowchart]. For any additional doses (i.e., the third dose and thereafter), patients in both groups will receive FP (in 1 U increments at the discretion of the ordering physician). No other aspects of care will be modified.

Figure 1. Study Flow Chart

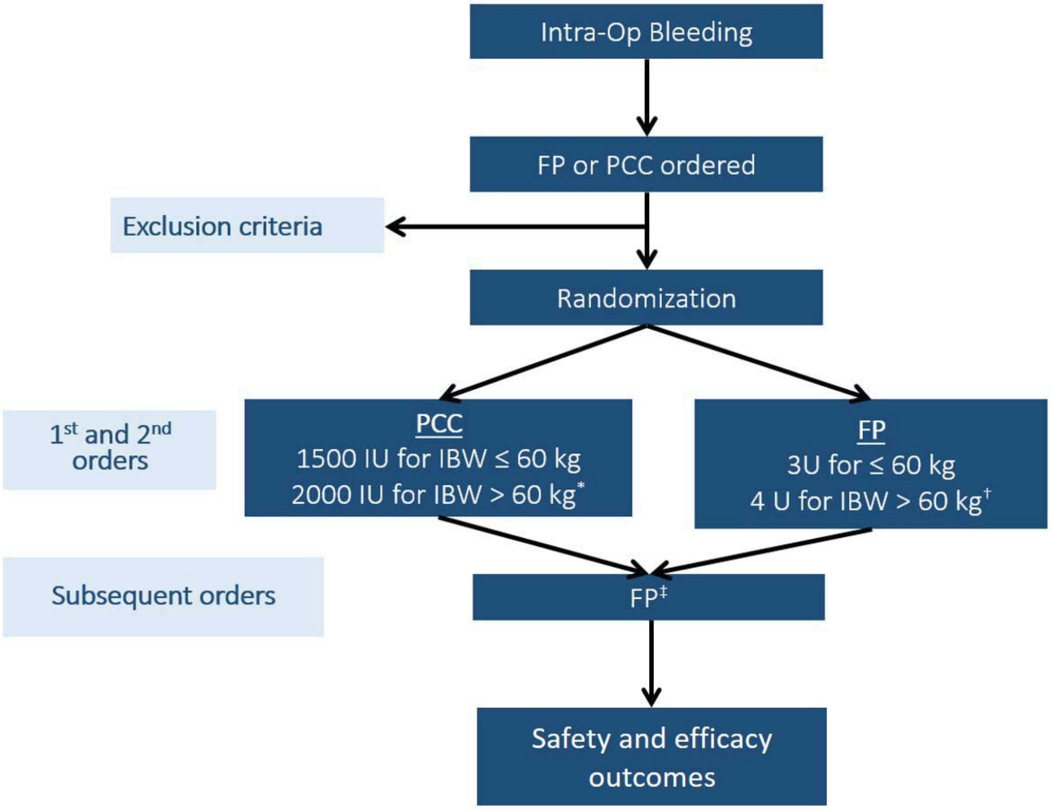

*PCC dose corresponds to a weight-based dosing range of 20–25 IU/kg_{IBW} rounded up to nearest 500 IU increment, up to a maximum of 2000 IU per dose

†FP dose corresponds to a weight-based dosing range of 10–15 mL/kg_{IBW} rounded up to the nearest 1 U increment, up to a maximum of 4 U per dose

‡FP in 1 U increments at the discretion of the ordering physician

PCC, prothrombin complex concentrate; FP, frozen plasma; IU, international units; RBC, red blood cell concentrate

1.3 Planned sample size

This pilot study will include 120 randomized patients, which, accounting for randomized but untreated patients and patients who withdraw in both arms, is expected to provide at least 100 evaluable patients, with 50 in each arm. This population size is expected to be sufficient to allow selection of an appropriate clinical endpoint for the confirmative Phase 3 study and to determine feasibility. No statistical sample size estimation has been involved.

2 GENERAL INFORMATION

2.1 Background details

All study data will be transferred to a SAS database (version 9.4 or later) for statistical analysis purposes. Data will be imported from an Electronic Data Capture System (OPVerdi) via validated SAS programs. If applicable, external data will also be transferred to SAS for presentation of these data in the statistical analyses.

The SAP will be finalized before any unblinded analysis after agreement with the Sponsor.

2.2 Deviations from the trial protocol with regard to statistical analyses

Overall, there were no deviations from the statistical methods stated in the protocol. However, to closer study attributable efficacy and safety results, additional exploratory analyses have been added, focusing explicitly on additional time windows. The additional time windows for these exploratory analyses are identified with italic font in section 5 below.

2.3 Individual protocol deviations

No specific protocol deviations will be considered as this is a non-interventional, pragmatic study.

A detailed review of all documented and derived deviations from protocol will be part of the Data Review Meeting (DMR) before database lock. During this DRM the impact of protocol deviations on the analysis will be assessed and the conclusions documented.

A complete listing of protocol deviations (documented as well as derived ones) and the judgment for assessment of subject disposition will be signed before clinical database lock. A description of protocol violations that led to exclusion from any analysis sets will be included in the table part of the Clinical Study Report (CSR).

3 ANALYSIS POPULATIONS

Due to the emergency nature of the condition being studied the trial will include only patients who are incapable of providing informed consent at the time the therapy is needed and in whom delays in obtaining surrogate consent can be severely detrimental to their well-being. Thus, this study qualifies for alteration to consent requirement before randomization. Therefore delayed consent from patients or surrogate decision maker (SDM) where appropriate will be sought after surgery.

As a rule, only data from enrolled patients with informed consent will be analyzed.

Data from randomized and treated patients who refused to give consent will be excluded from any analysis.

To ensure that the safety reporting is complete, all hemostatic therapy and severe or life-threatening serious adverse event data will be collected in cases where consent for remaining in the study cannot be obtained.

Hence, the disposition of patients will be displayed according to the following analysis populations:

- All Patients Enrolled (APE) population
- Failed Consent Patients (FCP) population
- Safety (SAF) population
- Intention-To-Treat (ITT) population
- Per-Protocol (PP) population

Membership of subjects will be decided upon in a DRM with the Sponsor before database lock. The proper flags for analysis sets exclusion (e.g., exclusion from PP set), will be included in the analysis datasets. The protocol deviation list should be finalized before database lock.

3.1 All Patients Enrolled (APE) Population

The APE population will include all enrolled patients regardless of the consent status who received at least one treatment with either PCC or FP. This population will only be used for displays of subject disposition.

3.2 Failed Consent Patients (FCP) Population

The FCP) population will consist of all enrolled patients who received at least one treatment with either PCC or FP for whom consent cannot be obtained. This population will only be used for displays of hemostatic therapy and severe or life-threatening serious adverse events.

3.3 Safety (SAF) Population

The Safety (SAF) population will include all enrolled patients who have given informed consent and who received at least one treatment with either PCC or FP regardless whether they underwent cardiac surgery or not. In the event that a patient receives treatment that is not in concordance with the randomization schedule, the treatment group will be defined according to the actual treatment received.

3.4 Intention-To-Treat (ITT) Population

The Intention-To-Treat (ITT) population will include all enrolled patients who have given informed consent and who received at least one treatment with either PCC or FP and underwent cardiac surgery. In the event that a patient receives treatment that is not in concordance with the randomization schedule, the treatment group will be defined according to the randomization (rather than the actual treatment received).

3.5 Per-Protocol (PP) Population

The Per-Protocol (PP) Population will include all patients of the IIT population, excluding patients with important protocol deviations that may affect the statistical analysis. The following patients will be excluded by default:

- Patients who receive an IMP different to the IMP assigned by randomization
- Patients who receive less than 80% of the planned dose
- Patients who significantly violate inclusion/exclusion criteria

3.6 Subgroup analyses

Due to the exploratory character of this pilot study efficacy and selected safety results will further be investigated within selected subgroups. This will be done to detect possible relationships that procedural or patient characteristics may have on the response parameters.

If the levels of subgroups (e.g. surgery types) are too numerous to form subgroups of sufficient size, the influence of the characteristic will be studied using model-based exploratory analyses (e.g. logistic regression [especially assessing the influence of IMP compliance on treatment response], Analysis of Covariance, Proportional hazards regression).

Presently, subgroup analyses are planned based on the following characteristics: sex; complexity of procedure (simple versus complex); and urgency of procedure (elective versus non-elective).

4 STATISTICAL ANALYSES

All statistical analyses will be performed using the SAS® software (Version 9.4 or later).

The analysis of safety will be based on the SAF and additionally on the ITT population.

The primary evaluation of overall efficacy will be performed on the ITT population. In addition selected efficacy analyses will also be presented for the PP population.

If not stated otherwise the following standard descriptive statistics will be presented:

– Descriptive statistics for continuous data

  N, mean, SD, min, lower quartile, median, upper quartile and max will be presented. These descriptive statistics will be determined for measured values and optionally for differences to baseline.

– Descriptive statistics for categorical data

  Absolute frequencies and percentages will be presented. Percentage bases (denominators) will be identified in the table title or footnote (i.e. all subjects at risk, all non-missing cases, all cases). For changes from baseline, shift tables may be generated.

– Exploratory statistics

Although statistical methods are primarily descriptive, two-sided 95% confidence intervals may be presented for selected parameters (e.g. incidences of adverse events) in an exploratory manner.

– Listings

All recorded data will be listed by subject. Identification variable will be the subject ID.

Derived data will be stored in special analysis data sets and will be calculated as outlined in section 6.1.

4.1 Conventions

4.1.1 Baseline definition

Baseline will be defined as the last value on or prior to the first IMP administration.

4.1.2 Missing data

No imputations for missing data will be performed.

4.1.3 Pooling of centers

No pooling of centers will be performed (only two centers are involved in the study).

4.2 Demographic and other background data

4.2.1 Basic description

All available demographic data (sex, age, height, weight, and Body Mass Index (BMI)) will be summarized in appropriate tables (summary statistics or frequency tables) for the ITT and PP populations.

4.3 IMP exposure, compliance

Dose and frequency of IMP administrations of PCC or FP will be summarized in appropriate tables (summary statistics or frequency tables) for the ITT and PP populations.

4.4 Medical history

Medical history findings will be coded using the MedDRA thesaurus in the version current at the time of study start. Coding will be performed by the CRO and agreed upon with the sponsor before database lock. (cf. DMP). The summary tables will display the medical history findings for pre-specified conditions by MedDRA System Organ class (SOC) and Preferred term (PT). Displays will be presented for the ITT and PP populations. Other medical history texts will only be listed.

4.5 Prior and concomitant medication

Medications will be coded using the WHO Drug Global thesaurus in the version current at the time of study start. Coding will be performed by the CRO and agreed upon with the sponsor

before database lock. (cf. DMP). For concomitant medications tables will show the frequencies of subjects by WHO preferred term. Prior medication will only be listed.

4.6 Efficacy

The primary population for the analysis of the efficacy of the IMPs will be the ITT. The primary efficacy endpoints will also be analyzed for the PP population.

The analysis of efficacy will focus on the following primary endpoints:

P1. Treatment response, defined as effective if no additional hemostatic intervention (such as administration of hemostatic agents including a second dose of IMP, platelet transfusion, or surgical re-exploration) in the time windows

- from 60 minutes to 4 and 24 hours after initiation of the first dose of IMP,
- *from initiation of the first dose of IMP to 4 and 24 hours.*

Otherwise, treatment response will be considered as not effective, see section 6.1. If the dose of IMP is repeated within less than 60 minutes, treatment response to the IMP will be assessed for the time frames after the second IMP dose only.

P2. Amount of allogeneic blood products (for each type of product and cumulatively) administered

- during the first 24 hours after the start of surgery,
- *during the first 24 hours after the end of CPB,*
- *during the first 24 hours after randomization,*
- *during the first 24 hours after the start of first IMP dose.*

P3. *N*umber of patients who do not receive any allogeneic blood product (in total and for each type separately)

- during the first 24 hours after the start of surgery,
- *during the first 24 hours after the end of CPB,*
- *during the first 24 hours after randomization,*
- *during the first 24 hours after the start of first IMP dose.*

In addition the following efficacy endpoints will be explored:

S1. Number of partial or full IMP doses of PCC and FP administered

- during the first 24 hours after the start of surgery.
- *during the first 24 hours after the end of CPB,*
- *during the first 24 hours after randomization,*
- *during the first 24 hours after the start of first IMP dose.*

S2. Incidence of major bleeding, using the universal definition of perioperative bleeding (UDPB) in cardiac surgery scores and their individual components

- during the first 24 hours after start of surgery,
- *during the first 24 hours after the end of CPB,*
- *during the first 24 hours after randomization,*
- *during the first 24 hours after the start of first IMP dose.*

as well as a modified version of the UDPB not including PCC and FP requirements.

S3. Amount of allogeneic blood products (for each type of product and cumulatively)

- from start of surgery to postoperative day 7,
- *from end of CPB to postoperative day 7,*
- *from randomization to postoperative day 7,*
- *from start of first IMP dose to postoperative day 7.*

S4. Number of patients who do not receive any allogeneic blood product (in total and for each type separately)

- from start of surgery to postoperative day 7.
- *from end of CPB to postoperative day 7,*
- *from randomization to postoperative day 7,*
- *from start of first IMP dose to postoperative day 7.*

S5. Number of patients receiving recombinant activated factor VII (rFVIIa)

- during the first 24 hours after the start of surgery
- *during the first 24 hours after the end of CPB,*
- *during the first 24 hours after randomization,*
- *during the first 24 hours after the start of first IMP dose.*

S6. Number of patients receiving fibrinogen concentrate

- during the first 24 hours after the start of surgery.
- *during the first 24 hours after the end of CPB,*
- *during the first 24 hours after randomization,*
- *during the first 24 hours after the start of first IMP dose.*

S7. Change in coagulation parameters (INR, ROTEM [EXTEM CT and MCF] and fibrinogen levels) within 75 minutes before and after IMP administration, where performed as part of standard of care.

S8. Time elapsed from first IMP administration to time leaving the operating room.

S9. Treatment response, defined as effective if no additional hemostatic intervention (such as administration of hemostatic agents including a second dose of IMP, platelet transfusion, or surgical re-exploration) in the time windows

- from 60 minutes to 4 and 24 hours after initiation of the first dose of IMP,
- *from initiation of the first dose of IMP to 4 and 24 hours.*

Otherwise, treatment response will be considered as not effective. If the dose of IMP is repeated within less than 60 minutes, treatment response to the IMP will be assessed as not effective.

Furthermore, the above mentioned efficacy endpoints will be exploratory analyzed in time frames which start at the time of first IMP dosing instead of the start of the surgery (as specified in section 2.2 of this SAP).

The following analysis methods will be applied to the different endpoints in according to their data type:

The analysis of categorical variables (P1, P3, S2, S4, S5, S6) will present relative frequency tables and estimates of the proportions with 95% confidence intervals separately in the treatment groups. The proportions will be compared between the treatment groups by means of Fisher's exact test.

The analysis of continuous variables (S7, S8) will present descriptive statistics of the sampling distribution and the differences between treatment groups. Also 95% confidence intervals and results of t-test of the treatment group differences will be reported.

The analysis of integer count variables (P2, S1, S3) will present descriptive statistics of the sampling distribution and the differences between treatment groups. Treatment group comparisons for variables (P2, S3) will be performed in the context of a counting regression model for the ABPs and their components (SAS PROC GENMOD with Negative-Binomial distribution). Tests and 95% confidence intervals for the treatment group differences will be derived from this statistical model and reported. The analysis for the sum of the allogenic blood products transfused (P2, S3) will be repeated analogously for the adjusted sum of the allogenic blood products transfused (cf. section 6.1), excluding FP.

4.7 Safety

All safety analyses will be based on the ITT population. If the SAF population differs from the ITT population then only AE information will additionally be displayed for the SAF.

The analysis of safety will study the following primary endpoints:

1. All adverse events (AEs) and serious AEs (SAEs) collected from beginning of surgery to postoperative day 28.

a. SAEs collected individually and as a composite (death, myocardial infarction, stroke, acute liver injury, acute kidney injury and thromboembolic events).

b. Transfusion-Related Acute Lung Injury (TRALI) and Transfusion-Associated Circulatory Overload (TACO)

4.7.1 Adverse events

Adverse events (AEs) will be coded according to the Medical Dictionary for Regulatory Activities (MedDRA). Coding will be agreed upon with the Sponsor before database lock (cf. DMP).

All adverse events recorded for patients in the SAF will be listed in the appendix of the study report differentiating by treatment emergent and non-treatment emergent events.

Only Treatment-emergent adverse events (TEAE) will be analyzed, i.e. all new and worsening pre-existing adverse events occurring after first IMP administration up to postoperative day 28. It is assumed that for each increase in intensity of an AE a new entry of the AE will be recorded by the investigator; hence such cases will be analyzed like different phases of the same AE.

A descriptive analysis will be performed. This analysis comprises the following set of tables separated by treatment group:

- Global incidence
- Incidences by primary system organ classes (SOC) and incidences of PT within primary SOC sorted according to the Internationally Agreed Order

Global incidences of primary system organ classes (SOC) and preferred terms (PT) will be calculated for

- All TEAE irrespective of the causality assessment
- TEAE by relationship (likely probable and possible related)
- TEAEs by worst severity
- Serious TEAEs

Multiple counts within a PT or SOC (repeated or different included terms or changes in descriptors) will be counted only once for the calculation of incidences.

A listing of 'special cases' containing subject identification, age, sex, AE descriptors, start and end of treatment will be prepared for the following types of TAEs:

- Fatal Serious adverse events (SAE)
- Adverse events which led to discontinuation
- Myocardial infraction
- Stroke
- Acute liver injury
- Acute kidney injury

- Thromboembolic events (according to MeDDRA SMQ)
- Transfusion-Related Acute Lung Injury (TRALI)
- Transfusion-Associated Circulatory Overload (TACO)

The number of patients who died will be summarized. A possible difference between treatment groups will be estimated by the risk ratio with 95% confidence interval. Kaplan-Meier estimates for the time to death distribution will be calculated and graphically presented.

4.7.2 Laboratory variables

In case of derived items in the database (e.g. after transformation to standard units, see DMP), only the derived items will be analyzed. Results of all individual lab tests will be listed in original and standard units in appendix 16.2 to the clinical trial report.

The following laboratory parameter will be analyzed (as available from local labs):

Coagulation profile	PT, PTT, INR, fibrinogen activity via Clauss assay,<br>ROTEM EXTEM CT, ROTEM EXTEM MCF
Hematology	Standard panel as per local lab
Clinical chemistry	Standard panel as per local lab
Safety labs	Troponin, ALT/ALP, Bilirubin, Creatinine

All laboratory values will be classified as normal or abnormal according to the laboratories' reference ranges and indicated as clinically significant or not clinically significant by the investigator on specified ranges. The following approaches will be taken for each laboratory parameter for the statistical analysis:

Quantitative data will be examined for trends using descriptive analysis (number of patients, number of missing values, mean, SD, median, quartiles, minimum, maximum) of actual values at each scheduled time point and changes from baseline to each scheduled time point. in addition mean concentration vs. time profiles (including standard deviations) will be plotted by treatment to illustrate any time trends.

Qualitative data based on reference ranges will be described according to the categories (i.e., low, normal, high).

Shift tables illustrating changes with respect to the laboratories' reference ranges between baseline and a defined scheduled time point.

Patient listings will be provided showing individual lab abnormalities.

4.8 Additional safety variables

The following additional safety variables will be summarized in tables separated by treatment group and the treatment differences be explored (exploratory tests and two-sided 95% confidence intervals):

1. Duration of mechanical ventilation up to postoperative day 28
2. Duration of intensive care unit (ICU) stay up to postoperative day 28

3. Duration of hospitalization up to postoperative day 28
4. Compliance of transfusion practice with the study's hemostatic algorithm. This will include comparison of lowest hemoglobin concentration
    - during the first 24 hours after the start of surgery,
    - *during the first 24 hours after end of CPB,*
    - *during the first 24 hours after randomization,*
    - *during the first 24 hours after the start of IMP*

4.9 Interim analyses

Not applicable.

5 QUALITY CONTROL

The SAP was reviewed by the Trial Statistician (TS) before signature. Particularly the TS has checked the consistency of the described methods and outputs with the actual version of the study protocol. In addition, a sponsor representative has reviewed the SAP before final approval.

Log files of all SAS® programs used in the analysis will be checked for errors, warnings and suspicious notes by the statistical programmer. All findings will be either eliminated or commented upon. The final version of each program will be stored along with its log file in the electronic archive.

All programs will be validated by the program author or an independent statistical programmer depending on the requested validation level selected in the List of TLFs.

The agreement of the program outputs with the SAP, their consistency and plausibility will be checked by the TS. Moreover, the TS will review the outputs regarding completeness, readability and comprehensibility.

The described process is associated with the 'normal' level of program validation. Additional levels of quality control can be specified in the List of TLFs (see Appendix, 1) for individual outputs.

6 DERIVATIONS AND TRANSFORMATIONS

6.1 Formulas for derived variables

Variable	Definition / Derivation
Response (4h)<br>(Treatment response)	= 'Effective' ('Yes') if no additional hemostatic intervention occurred (such as administration of hemostatic agents including a second dose of IMP, platelet transfusion, or surgical re-exploration) from 60 minutes to 4 hours after initiation of the first dose of IMP.<br>= 'Ineffective' ('No') otherwise.<br>If the dose of IMP is repeated within less than 60 minutes, treatment response to the IMP will be assessed for the 60 minutes after the second IMP dose only.
Response (24h)<br>(Treatment response)	= 'Effective' ('Yes') if no additional hemostatic intervention occurred (such as administration of hemostatic agents including a second dose of IMP, platelet transfusion, or surgical re-exploration) from 60 minutes to 24 hours after initiation of the first dose of IMP.<br>= 'Ineffective' ('No') otherwise.<br>If the dose of IMP is repeated within less than 60 minutes, treatment response to the IMP will be assessed for the 60 minutes after the second IMP dose only.
ABP(time frame)	= Sum of all Allogenic Blood Products (RBCs, FPs and Platelets) transfused in specified time frame
ABP_{adj}(time frame)	= Adjusted Sum of Allogenic Blood Products (RBCs and Platelets) transfused in specified time frame, I.e. ABP excluding FPs

6.2 Transformations to be applied

In order that the sim of Allogenic Blood Products and its constituents adequately represent the exposure to donors the following transformations will be applied before any derivations:
Platelets:
1 apheresis unit = 4 allogeneic units,
1 non-apheresis unit = 4 allogeneic units
Frozen Plasma (used in transfusions):
1 apheresis unit = 2 allogeneic units,
1 non-apheresis unit = 1 allogeneic unit

Transfusion units without any information about apheresis are handled as apheresis units.

7 REFERENCES

No specific references were used.

APPENDICES

1. List of Tables, Listings, Figures

A complete List of tables, listings, figures (TLFs) will be given in a separate document which can be updated without updating the SAP. The List will serve as a reference for the Sponsor, the TS and the statistical programmer and describes the entire set of statistical output to be produced. Therefore, this List will be versioned and approved by both Ergomed and Sponsor before commencing the statistical programming.

Each output page will have an appropriate heading specifying the study ID and abbreviated study title.

Each output page will show a common date and page numbers in the form 'Page [x / y]' where x denotes the current page within an output and y the total number of pages of that output. The output pages will not contain any other sequential page numbering.

All statistical output will identify the underlying analysis set(s) and indicate the number of subjects/events in this set (N) and the number of subjects/events actually contributing to the particular output (n).

All subject listings will contain in addition to the subject identification the treatment arm and the analysis set.
