# Supplementary material for: Comparison of 4-Factor Prothrombin Complex Concentrate With Frozen Plasma for Management of Hemorrhage During and After Cardiac Surgery: A Randomized Pilot Trial
Source: JAMA Netw Open. 2021 Apr 1;4(4):e213936. doi: 10.1001/jamanetworkopen.2021.3936 (PMC8017469; doi:10.1001/jamanetworkopen.2021.3936)
Supplement: Supplement 2. — Data Sharing Statement [file jamanetwopen-e213936-s002.pdf]

Data Sharing Statement

Karkouti. Comparison of 4-Factor Prothrombin Complex Concentrate With Frozen Plasma for Management of Hemorrhage During and After Cardiac Surgery. *JAMA Netw Open*. Published April 01, 2021. doi:10.1001/jamanetworkopen.2021.3936

Data
Data available: No
